# Looking for adaptive footprints in the *HSP90AA1* ovine gene

**DOI:** 10.1186/s12862-015-0280-x

**Published:** 2015-02-04

**Authors:** Judit Salces-Ortiz, Carmen González, Marta Martínez, Tomás Mayoral, Jorge H Calvo, M Magdalena Serrano

**Affiliations:** INIA, Carretera de La Coruña Km. 7,5. 280040, Madrid, Spain; Laboratorio Central de Veterinaria, MAGRAMA, Ctra. M-106 km1.4 28110, Madrid, Spain; CITA, Avda, Montañana, 930, 50059, Zaragoza, Spain

**Keywords:** Sheep breeds, Climatic variables, *HSP90AA1* polymorphisms, Bayesian test, Caprinae species

## Abstract

**Background:**

Climatic factors play an important role in determining species distributions and phenotypic variation of populations over geographic space. Since domestic sheep is managed under low intensive systems animals could have retained some genome adaptive footprints. The gene encoding the Hsp90α has been extensively studied in sheep and some polymorphisms located at its promoter have been associates with differences in the transcription rate of the gene depending on climatic conditions. In this work the relationships among the distribution and frequencies of 11 polymorphisms of the ovine *HSP90AA1* gene promoter in 31 sheep breeds and the climatic and geographic variables prevailing in their regions of origin have been studied. Also the promoter sequence has been characterized in 9 species of the Caprinae subfamily*.*

**Results:**

Correlations among several climatic variables and allele frequencies of the polymorphisms of the *HSP90AA1* gene promoter linked with differences in the transcription activity of the gene under heat stress conditions have been assessed. A group of breeds reared in semi dry climates have high frequencies of the insertion allele of the g.667-668insC associated with the heat stress response. Other group of breeds native to semi arid conditions showed very low frequencies of this same allele. However, in some cases, this previous correlation has not been achieved, revealing the high levels of gene flow among populations occurred following domestication. The Bayesian Test of Beaumont and Balding identified two outlier loci, the g.522A > G and g.703_704del(2)A candidates to balancing and directional selection, respectively. Polymorphisms detected in *O. aries* are also present in several species of the Caprinae subfamily being *C. hircus*, *O. musimon* and *O. moschatus* those sharing the highest number of them with *O. aries*.

**Conclusions:**

Despite domestication, sheep breeds showed some genetic footprints related to climatic variables. Adaptation of breeds to heat climates can suppose a selective advantage to cope with global warming caused by climatic change. Polymorphisms of the *HSP90AA1* gene detected in the *Ovis aries* species are also present in wild species from the Caprinae subfamily, indicating a great antiquity of these mutations and its importance in the adaptation of species to past climatic conditions existing in its native environments.

**Electronic supplementary material:**

The online version of this article (doi:10.1186/s12862-015-0280-x) contains supplementary material, which is available to authorized users.

## Background

The Subfamily Caprinae includes a widespread and diverse group of ungulates (hoofed mammals) that are most extending from the arctic to the equator. Wild Caprinae were the ancestors of two of the most important species of domestic livestock - domestic sheep (*Ovis aries*) and goats (*Capra hircus*). Present day populations of wild Caprinae represent a potential source of knowledge of adaptation genetics which can be used to improve or adapt current domestic breeds to less productive conditions [1].

Sheep was one of the first species to be domesticated, approximately 11,000 years before the present in the Fertile Crescent [2], due to its small size, docile behavior and high adaptability to very different environments. This domestication process must have involved a genetically broad sampling of wild stock and also the persistence of cross-breeding with wild populations [3]. Domestication pressure over animal’s life had as consequence that natural selection loosed impact over their biological fitness giving up the turn to artificial selection imposed by humans over productive traits (wool, meat, milk). However, sheep is one of the livestock species managed under low intensive systems and therefore could have retained from its wild ancestors some genome footprints in genes related to environmental adaptation.

Climatic factors like temperature and humidity play an important role in determining species distributions and they likely influence phenotypic variation of populations over geographic space [4]. Correlations between phenotype and environment may be revealed by genetic polymorphisms which allele frequencies strongly differentiate populations that live in different environments [5] and such differences can be maintained in the face of gene flow [6].

Several studies have examined the distributions of genetic variants in candidate genes for traits that vary with climate. For example, in humans, candidate gene studies have yielded evidence that variants involved in sodium homeostasis and energy metabolism [4] and those related with type 2 diabetes and obesity [7] are strongly correlated with climate variables. Also a decrease in the frequency of variants implicated in salt sensitive hypertension had been correlated with increasing distance from the equator [8]. In *Drosophila melanogaster*, variants involved in circadian rhythms, aging and energy metabolism were correlated with climate [9], in *Arabidopsis thaliana*, variants associated with flowering time were correlated with latitude [10], and in pines several genes contain variation have been correlated with temperature [11].

The heat shock response is among the most important and ubiquitous fact in nature. Heat, both quantitatively and qualitatively is one of the best inducers of Heat Stress Proteins (Hsp). They act as molecular chaperones, helping to maintain the metabolic and structural integrity of the cell, as a protective response to external stresses. The chaperone Hsp90 is one of the most abundant, highly conserved and usually heat-induced proteins found in all eukaryotes studied so far. *HSP90* gene presents two isoforms, *HSP90-α* (inducible form) and *HSP90- β* (constitutive form). There are only few publications on the role of Hsp90 function in species adaptation and survival under extreme conditions [12-14]. The gene encoding the Hsp90α heat-shock protein (*HSP90AA1*) has been extensively studied in sheep [15-18]. Differences in the *HSP90AA1* transcription rate [18-20] depending on genotype combination of some polymorphisms located at its promoter and the environmental conditions existing when sample collections have been shown. Also an effect of these polymorphisms over ram’s sperm DNA fragmentation depending on environmental temperatures has been assessed [20,21].

This work has the aim to study the relationships between the frequencies of 11 polymorphisms located in the *HSP90AA1* gene promoter in 31 sheep breeds from different locations of the European, Asian and Africa continents and the climatic and geographic variables prevailing in the regions where these breeds are reared; and to characterize the *HSP90AA1* promoter sequence in 9 species of the Caprinae and in 2 species of the Bovinae subfamilies to determine polymorphisms history and contribute to elucidate the phylogeny of one of the most controversial subfamily of the sub order Ruminantia.

## Results

### Polymorphism variability and test for linkage disequilibrium in sheep breeds

Genotype and allele frequencies of the 11 polymorphisms studied in each of the 31 sheep breeds are showed in Tables 1 and 2. Levels of polymorphism were generally high in all breeds. There were no private alleles in any of the breeds studied. The less polymorphic marker was the SNP g.522 > G for which the G allele was fixed in 18 breeds. For the INDELs g.666_667insC and g.516_517insG, the D allele was fixed in nine and six breeds, respectively.

**Table 1:** **Genotype frequencies of the 11 polymorphisms located at the** ***HSP90AA1*** **gene in the 31 sheep breeds studied**

		**g.703_704del(2)A**			**g.667_668insC**			**g.666_667insC**			**g.660G > C**		**g.601A > C**				**g.528G > A**			**g.524G > T**			**g.522A > G**			**g.516_517insG**			**g.468G > T**			**g.444A > G**		
**Breed**	**ID breed**	**DD**	**AD**	**AA**	**II**	**ID**	**DD**	**II**	**ID**	**DD**	**GG**	**CG**	**CC**	**AA**	**AC**	**CC**	**GG**	**AG**	**AA**	**GG**	**GT**	**TT**	**AA**	**AG**	**GG**	**II**	**ID**	**DD**	**GG**	**GT**	**TT**	**AA**	**AG**	**GG**
Akkaraman	AKA	0.13	0.52	0.35	0.00	0.48	0.52	0.04	0.22	0.74	0.13	0.52	0.35	0.04	0.39	0.57	0.04	0.57	0.39	0.00	0.39	0.61	0.00	0.09	0.91	0.00	0.04	0.96	0.00	0.39	0.61	0.00	0.26	0.74
Kazakh Arkhar-Merino	ARME	0.11	0.50	0.39	0.00	0.50	0.50	0.17	0.00	0.83	0.06	0.50	0.44	0.00	0.17	0.83	0.06	0.44	0.50	0.00	0.17	0.83	0.00	0.00	1.00	0.00	0.39	0.61	0.00	0.17	0.83	0.00	0.28	0.72
Assaf	AS	0.00	0.20	0.80	0.13	0.53	0.33	0.10	0.10	0.80	0.00	0.20	0.80	0.00	0.57	0.43	0.00	0.20	0.80	0.00	0.57	0.43	0.00	0.00	1.00	0.00	0.00	1.00	0.00	0.57	0.43	0.03	0.17	0.80
Awassi	AW	0.00	0.30	0.70	0.03	0.49	0.49	0.00	0.00	1.00	0.00	0.27	0.73	0.13	0.57	0.30	0.00	0.27	0.73	0.13	0.57	0.30	0.00	0.00	1.00	0.00	0.00	1.00	0.13	0.57	0.30	0.00	0.03	0.97
Bajdarak	BAJ	0.27	0.50	0.23	0.00	0.14	0.86	0.00	0.00	1.00	0.14	0.64	0.23	0.00	0.36	0.64	0.05	0.59	0.36	0.00	0.36	0.64	0.00	0.14	0.86	0.00	0.05	0.95	0.00	0.32	0.68	0.00	0.18	0.82
Bni Guil	BNI	0.15	0.56	0.30	0.00	0.37	0.63	0.04	0.22	0.74	0.15	0.56	0.30	0.00	0.22	0.78	0.11	0.48	0.41	0.00	0.15	0.85	0.00	0.11	0.89	0.00	0.15	0.85	0.04	0.19	0.78	0.04	0.26	0.70
Boujaad	BOUJ	0.17	0.25	0.58	0.00	0.46	0.54	0.00	0.13	0.88	0.17	0.25	0.58	0.00	0.25	0.75	0.00	0.33	0.58	0.00	0.00	0.01	0.00	0.13	0.88	0.00	0.21	0.79	0.00	0.25	0.75	0.00	0.17	0.83
Bozakh	BOZ	0.33	0.29	0.38	0.04	0.38	0.58	0.08	0.13	0.79	0.33	0.29	0.38	0.00	0.21	0.79	0.17	0.46	0.38	0.00	0.21	0.79	0.00	0.00	1.00	0.00	0.08	0.92	0.00	0.21	0.79	0.04	0.17	0.79
Caucasian	CAUC	0.20	0.32	0.48	0.04	0.32	0.64	0.12	0.00	0.88	0.12	0.40	0.48	0.00	0.20	0.80	0.08	0.44	0.48	0.00	0.12	0.88	0.00	0.00	1.00	0.12	0.32	0.56	0.00	0.20	0.80	0.04	0.16	0.80
Churra	Ch	0.00	0.52	0.48	0.09	0.30	0.61	0.04	0.26	0.70	0.00	0.52	0.48	0.00	0.26	0.74	0.00	0.52	0.48	0.00	0.26	0.74	0.00	0.22	0.78	0.00	0.30	0.70	0.00	0.26	0.74	0.04	0.35	0.61
Churra Lebrijana	Cl	0.00	0.42	0.58	0.38	0.42	0.19	0.00	0.00	1.00	0.00	0.38	0.62	0.00	0.12	0.88	0.00	0.38	0.62	0.00	0.15	0.85	0.00	0.00	1.00	0.00	0.04	0.96	0.00	0.15	0.85	0.00	0.04	0.96
Churra Tensina	Ct	0.24	0.36	0.39	0.00	0.18	0.82	0.00	0.00	1.00	0.15	0.42	0.42	0.03	0.00	0.97	0.15	0.42	0.42	0.00	0.03	0.97	0.00	0.00	1.00	0.09	0.45	0.45	0.00	0.03	0.97	0.00	0.03	0.97
Daglic	DGL	0.42	0.42	0.17	0.00	0.08	0.92	0.00	0.00	1.00	0.42	0.42	0.17	0.13	0.13	0.75	0.42	0.42	0.17	0.13	0.13	0.75	0.00	0.00	1.00	0.00	0.04	0.96	0.13	0.13	0.75	0.00	0.00	1.00
Kazakh Edilbai	EDIL	0.00	0.10	0.90	0.00	0.03	0.97	0.00	0.00	1.00	0.33	0.67	0.00	0.00	0.03	0.97	0.27	0.70	0.03	0.00	0.03	0.97	0.00	0.03	0.97	0.00	0.10	0.90	0.00	0.00	0.01	0.00	0.00	1.00
Ivesi	IV	0.20	0.27	0.53	0.07	0.13	0.80	0.07	0.07	0.87	0.20	0.27	0.53	0.13	0.33	0.53	0.20	0.20	0.60	0.13	0.33	0.53	0.00	0.07	0.93	0.00	0.00	1.00	0.13	0.33	0.53	0.07	0.07	0.87
Russian Karakul	KAR	0.27	0.73	0.00	0.00	0.00	1.00	0.00	0.00	1.00	0.20	0.80	0.00	0.00	0.60	0.40	0.27	0.73	0.00	0.00	0.60	0.40	0.00	0.00	1.00	0.00	0.00	1.00	0.07	0.53	0.40	0.00	0.00	1.00
Moldavian Karakul	KARM	0.73	0.27	0.00	0.07	0.00	0.93	0.00	0.00	1.00	0.27	0.73	0.00	0.00	0.40	0.60	0.27	0.67	0.07	0.00	0.40	0.60	0.00	0.00	1.00	0.00	0.07	0.93	0.00	0.40	0.60	0.00	0.07	0.93
Karabakh	KRB	0.25	0.67	0.08	0.00	0.42	0.58	0.00	0.08	0.92	0.25	0.67	0.08	0.00	0.17	0.83	0.13	0.71	0.17	0.00	0.17	0.83	0.00	0.08	0.92	0.00	0.00	1.00	0.00	0.25	0.75	0.00	0.25	0.75
Karachai	KRC	0.36	0.54	0.11	0.00	0.25	0.75	0.04	0.07	0.89	0.25	0.61	0.14	0.00	0.29	0.71	0.25	0.61	0.14	0.00	0.29	0.71	0.00	0.04	0.96	0.00	0.04	0.96	0.00	0.29	0.71	0.00	0.18	0.82
Karayaka	KRY	0.09	0.36	0.55	0.00	0.18	0.82	0.05	0.05	0.91	0.09	0.36	0.55	0.09	0.32	0.59	0.09	0.27	0.64	0.05	0.36	0.59	0.00	0.05	0.95	0.00	0.27	0.73	0.09	0.36	0.55	0.00	0.14	0.86
Kivircik	KVR	0.00	0.50	0.50	0.13	0.63	0.25	0.00	0.13	0.88	0.00	0.50	0.50	0.00	0.19	0.81	0.06	0.31	0.63	0.00	0.13	0.88	0.00	0.00	1.00	0.00	0.06	0.94	0.00	0.19	0.81	0.00	0.19	0.81
Finnsheep	L	0.17	0.50	0.33	0.10	0.37	0.53	0.10	0.37	0.53	0.17	0.50	0.33	0.03	0.03	0.93	0.10	0.47	0.43	0.03	0.03	0.93	0.00	0.00	1.00	0.00	0.03	0.97	0.03	0.03	0.93	0.10	0.37	0.53
Latxa	LX	0.02	0.46	0.51	0.00	0.49	0.51	0.00	0.37	0.63	0.00	0.22	0.78	0.00	0.29	0.71	0.00	0.20	0.80	0.00	0.29	0.71	0.00	0.00	1.00	0.02	0.37	0.61	0.00	0.29	0.71	0.00	0.46	0.54
D’Man	MAN	0.08	0.46	0.46	0.00	0.08	0.92	0.00	0.08	0.92	0.04	0.50	0.46	0.08	0.38	0.54	0.00	0.04	0.96	0.08	0.31	0.62	0.00	0.00	1.00	0.08	0.27	0.65	0.08	0.38	0.54	0.12	0.38	0.50
Spanish Merino	ME	0.17	0.31	0.52	0.07	0.48	0.45	0.03	0.10	0.86	0.07	0.41	0.52	0.00	0.14	0.86	0.03	0.21	0.76	0.00	0.14	0.86	0.00	0.10	0.90	0.00	0.17	0.83	0.00	0.14	0.86	0.03	0.41	0.55
Manchega	MNCH	0.05	0.53	0.42	0.02	0.42	0.57	0.00	0.07	0.93	0.05	0.53	0.42	0.00	0.33	0.67	0.03	0.53	0.43	0.00	0.32	0.68	0.00	0.03	0.97	0.02	0.33	0.65	0.00	0.32	0.68	0.00	0.10	0.90
Olkuska	OL	0.10	0.47	0.43	0.03	0.50	0.47	0.07	0.37	0.57	0.10	0.47	0.43	0.00	0.00	1.00	0.10	0.47	0.43	0.00	0.00	1.00	0.00	0.00	1.00	0.00	0.17	0.83	0.00	0.00	1.00	0.07	0.43	0.50
Pramenka	PRAM	0.21	0.24	0.55	0.14	0.41	0.45	0.10	0.34	0.55	0.21	0.24	0.55	0.00	0.31	0.69	0.21	0.21	0.59	0.00	0.31	0.69	0.00	0.00	1.00	0.00	0.10	0.90	0.00	0.31	0.69	0.03	0.48	0.48
Rasa Aragonesa	RA	0.05	0.33	0.62	0.07	0.40	0.52	0.07	0.07	0.86	0.05	0.31	0.64	0.02	0.17	0.81	0.05	0.24	0.71	0.02	0.17	0.81	0.00	0.10	0.90	0.05	0.31	0.64	0.02	0.17	0.81	0.05	0.19	0.76
Sakiz	SZ	0.04	0.38	0.58	0.19	0.38	0.42	0.00	0.00	1.00	0.04	0.38	0.58	0.00	0.12	0.88	0.00	0.31	0.69	0.00	0.12	0.88	0.00	0.00	1.00	0.00	0.00	1.00	0.00	0.08	0.92	0.00	0.00	1.00
Valle del Belice	VdB	0.14	0.55	0.31	0.03	0.31	0.66	0.10	0.03	0.86	0.10	0.55	0.34	0.00	0.00	1.00	0.07	0.45	0.48	0.00	0.03	0.97	0.00	0.00	1.00	0.03	0.31	0.66	0.00	0.03	0.97	0.03	0.21	0.76

**Table 2:** **Allele frequencies of the 11 polymorphisms located at the** ***HSP90AA1*** **gene in the 31 sheep breeds studied**

			**g.703_704del(2)A**		**g.667_668insC**		**g.666_667insC**		**g.660G > C**		**g.601A > C**		**g.528G > A**		**g.524G > T**		**g.522ª > G**		**g.516_517insG**		**g.468G > T**		**g.444A > G**	
**Breed**	**ID breed**	**N**	**D**	**AA**	**I**	**D**	**I**	**D**	**G**	**C**	**A**	**C**	**G**	**A**	**G**	**T**	**A**	**G**	**I**	**D**	**G**	**T**	**A**	**G**
Akkaraman	AKA	46	0.39	0.61	0.24	0.76	0.24	0.85	0.39	0.61	0.24	0.76	0.33	0.67	0.20	0.80	0.04	0.96	0.02	0.98	0.20	0.80	0.13	0.87
Kazakh Arkhar-Merino	ARME	36	0.36	0.64	0.25	0.75	0.08	0.83	0.31	0.69	0.08	0.92	0.28	0.72	0.08	0.92	0.00	1.00	0.19	0.81	0.08	0.92	0.14	0.86
Assaf	AS	60	0.10	0.90	0.40	0.60	0.15	0.85	0.10	0.90	0.28	0.72	0.10	0.90	0.28	0.72	0.00	1.00	0.00	1.00	0.28	0.72	0.12	0.88
Awassi	AW	60	0.15	0.85	0.27	0.73	0.00	1.00	0.13	0.87	0.42	0.58	0.13	0.87	0.42	0.58	0.00	1.00	0.00	1.00	0.42	0.58	0.02	0.98
Bajdarak	BAJ	44	0.52	0.48	0.07	0.93	0.00	1.00	0.45	0.55	0.18	0.82	0.34	0.66	0.18	0.82	0.07	0.93	0.02	0.98	0.16	0.84	0.09	0.91
Bni Guil	BNI	54	0.43	0.57	0.19	0.81	0.24	0.85	0.43	0.57	0.11	0.89	0.35	0.65	0.07	0.93	0.06	0.94	0.07	0.93	0.13	0.87	0.17	0.83
Boujaad	BOUJ	48	0.46	0.54	0.23	0.77	0.13	0.94	0.46	0.54	0.13	0.88	0.25	0.75	0.13	0.88	0.06	0.94	0.10	0.90	0.13	0.88	0.08	0.92
Bozakh	BOZ	48	0.48	0.52	0.23	0.77	0.17	0.85	0.48	0.52	0.10	0.90	0.40	0.60	0.10	0.90	0.00	1.00	0.04	0.96	0.10	0.90	0.13	0.88
Caucasian	CAUC	50	0.36	0.64	0.20	0.80	0.06	0.88	0.32	0.68	0.10	0.90	0.30	0.70	0.06	0.94	0.00	1.00	0.28	0.72	0.10	0.90	0.12	0.88
Churra	Ch	46	0.26	0.74	0.24	0.76	0.28	0.83	0.26	0.74	0.13	0.87	0.26	0.74	0.13	0.87	0.11	0.89	0.15	0.85	0.13	0.87	0.22	0.78
Churra Lebrijana	Cl	52	0.21	0.79	0.60	0.40	0.00	1.00	0.19	0.81	0.06	0.94	0.19	0.81	0.08	0.92	0.00	1.00	0.02	0.98	0.08	0.92	0.02	0.98
Churra Tensina	Ct	66	0.42	0.58	0.09	0.91	0.00	1.00	0.36	0.64	0.03	0.97	0.36	0.64	0.02	0.98	0.00	1.00	0.32	0.68	0.02	0.98	0.02	0.98
Daglic	DGL	48	0.63	0.38	0.04	0.96	0.00	1.00	0.63	0.38	0.19	0.81	0.63	0.38	0.19	0.81	0.00	1.00	0.02	0.98	0.19	0.81	0.00	1.00
Kazakh Edilbai	EDIL	60	0.95	0.05	0.02	0.98	0.00	1.00	0.67	0.33	0.02	0.98	0.62	0.38	0.02	0.98	0.02	0.98	0.05	0.95	0.02	0.98	0.00	1.00
Ivesi	IV	30	0.47	0.53	0.13	0.87	0.10	0.90	0.47	0.53	0.30	0.70	0.30	0.70	0.30	0.70	0.03	0.97	0.00	1.00	0.30	0.70	0.10	0.90
Russian Karakul	KAR	30	0.63	0.37	0.00	1.00	0.00	1.00	0.60	0.40	0.30	0.70	0.63	0.37	0.30	0.70	0.00	1.00	0.00	1.00	0.33	0.67	0.00	1.00
Moldavian Karakul	KARM	30	0.87	0.13	0.07	0.93	0.00	1.00	0.63	0.37	0.20	0.80	0.60	0.40	0.20	0.80	0.00	1.00	0.03	0.97	0.20	0.80	0.03	0.67
Karabakh	KRB	48	0.58	0.42	0.21	0.79	0.08	0.96	0.58	0.42	0.08	0.92	0.48	0.52	0.08	0.92	0.04	0.96	0.00	1.00	0.13	0.88	0.13	0.88
Karachai	KRC	56	0.63	0.38	0.13	0.88	0.09	0.93	0.55	0.45	0.14	0.86	0.55	0.45	0.14	0.86	0.02	0.98	0.02	0.98	0.14	0.86	0.09	0.91
Karayaka	KRY	44	0.27	0.73	0.09	0.91	0.07	0.93	0.27	0.73	0.25	0.75	0.23	0.77	0.23	0.77	0.02	0.98	0.14	0.86	0.27	0.73	0.07	0.93
Kivircik	KVR	32	0.25	0.75	0.44	0.56	0.13	0.94	0.25	0.75	0.09	0.91	0.22	0.78	0.06	0.94	0.00	1.00	0.03	0.97	0.09	0.91	0.09	0.91
Finnsheep	L	60	0.42	0.58	0.28	0.72	0.42	0.72	0.42	0.58	0.05	0.95	0.33	0.67	0.05	0.95	0.00	1.00	0.02	0.98	0.05	0.95	0.28	0.72
Latxa	LX	82	0.28	0.72	0.24	0.76	0.37	0.82	0.11	0.89	0.15	0.85	0.10	0.90	0.15	0.85	0.00	1.00	0.21	0.79	0.15	0.85	0.23	0.77
D’Man	MAN	52	0.31	0.69	0.04	0.96	0.08	0.96	0.29	0.71	0.27	0.73	0.02	0.98	0.23	0.77	0.00	1.00	0.21	0.79	0.27	0.73	0.31	0.69
Spanish Merino	ME	58	0.33	0.67	0.31	0.69	0.12	0.91	0.28	0.72	0.07	0.93	0.14	0.86	0.07	0.93	0.05	0.95	0.09	0.91	0.07	0.93	0.24	0.76
Manchega	MNCH	120	0.32	0.68	0.23	0.78	0.07	0.97	0.32	0.68	0.17	0.83	0.30	0.70	0.16	0.84	0.02	0.98	0.18	0.82	0.16	0.84	0.05	0.95
Olkuska	OL	60	0.33	0.67	0.28	0.72	0.40	0.75	0.33	0.67	0.00	1.00	0.33	0.67	0.00	1.00	0.00	1.00	0.08	0.92	0.00	1.00	0.28	0.72
Pramenka	PRAM	58	0.33	0.67	0.34	0.66	0.40	0.72	0.33	0.67	0.16	0.84	0.31	0.69	0.16	0.84	0.00	1.00	0.05	0.95	0.16	0.84	0.28	0.72
Rasa Aragonesa	RA	84	0.21	0.79	0.27	0.73	0.11	0.89	0.20	0.80	0.11	0.89	0.17	0.83	0.11	0.89	0.05	0.95	0.20	0.80	0.11	0.89	0.14	0.86
Sakiz	SZ	52	0.23	0.77	0.38	0.62	0.00	1.00	0.23	0.77	0.06	0.94	0.15	0.85	0.06	0.94	0.00	1.00	0.00	1.00	0.04	0.96	0.00	1.00
Valle del Belice	VdB	58	0.41	0.59	0.19	0.81	0.09	0.88	0.38	0.62	0.00	1.00	0.29	0.71	0.02	0.98	0.00	1.00	0.19	0.81	0.02	0.98	0.14	0.86

It is outstanding that seven polymorphisms had the MAF for the same allele in all breeds (I_-668_, I_-667_, A_-601_, G_-524_, A_-522_, I_-516_, G_-468_ and A_-444_). However, the MAF for g.703_704del(2)A, g.660G > C and g.528G > A polymorphisms were the AA_-704_, C_-660_, and A_-528_ alleles in five Asian (DGL, EDIL, KAR, KRB and KRC) and one European (KARM) breeds, while for the remaining breeds were the D_-704_, G_-660_ and G_-528_ alleles (Tables 1 and 2).

The Hardy Weinberg equilibrium test for all breeds joined (Additional file 1, AF1) shows all polymorphisms in HW equilibrium except for the INDELs g.666_667insC and g.703_704del(2)A. The average expected (Ehet) and observed (Ohet) heterozygosis were 0.273 and 0.258, respectively, for all breeds joined.

Linkage disequilibrium (LD) was estimated to obtain polymorphism linked blocks across and within breeds. Additional file 2 (AF2) shows the LD matrix for all populations and for each breed separately and also a figure of LD blocks and haplotypes. In most breeds, similar LD than those previously observed in Manchega Spanish breed (MNCH) [19] were obtained. Thus, three LD blocks of polymorphisms can be established: g.666_667insC_g.444A > G; g.703_704del(2)A_g.660G > C_g.528A > G and g.601A > C_g.524G > T_g.468G > T.

### Phylogenetic relationships between sheep breeds

Additional file 3 (AF3) shows population pairwise F_STs_, p values and significances and the Reynolds’s distance matrix among the 31 sheep breeds studied. Average, median, maximum and minimum distances across populations were 0.0952, 0.0628, 0.6159 and 0.0000, respectively. Among AW, SZ, AS, Cl, LX, KAR, DGL, KARM and EDIL breeds distances higher than 0.25 were observed. Breeds with distance values lower than 0.01 (even 0.00) among them were found for ARME, Ch, KRY, MNCH, AKA, CAUC, BNI, BOUJ and BOZ.

**Figure 1:**
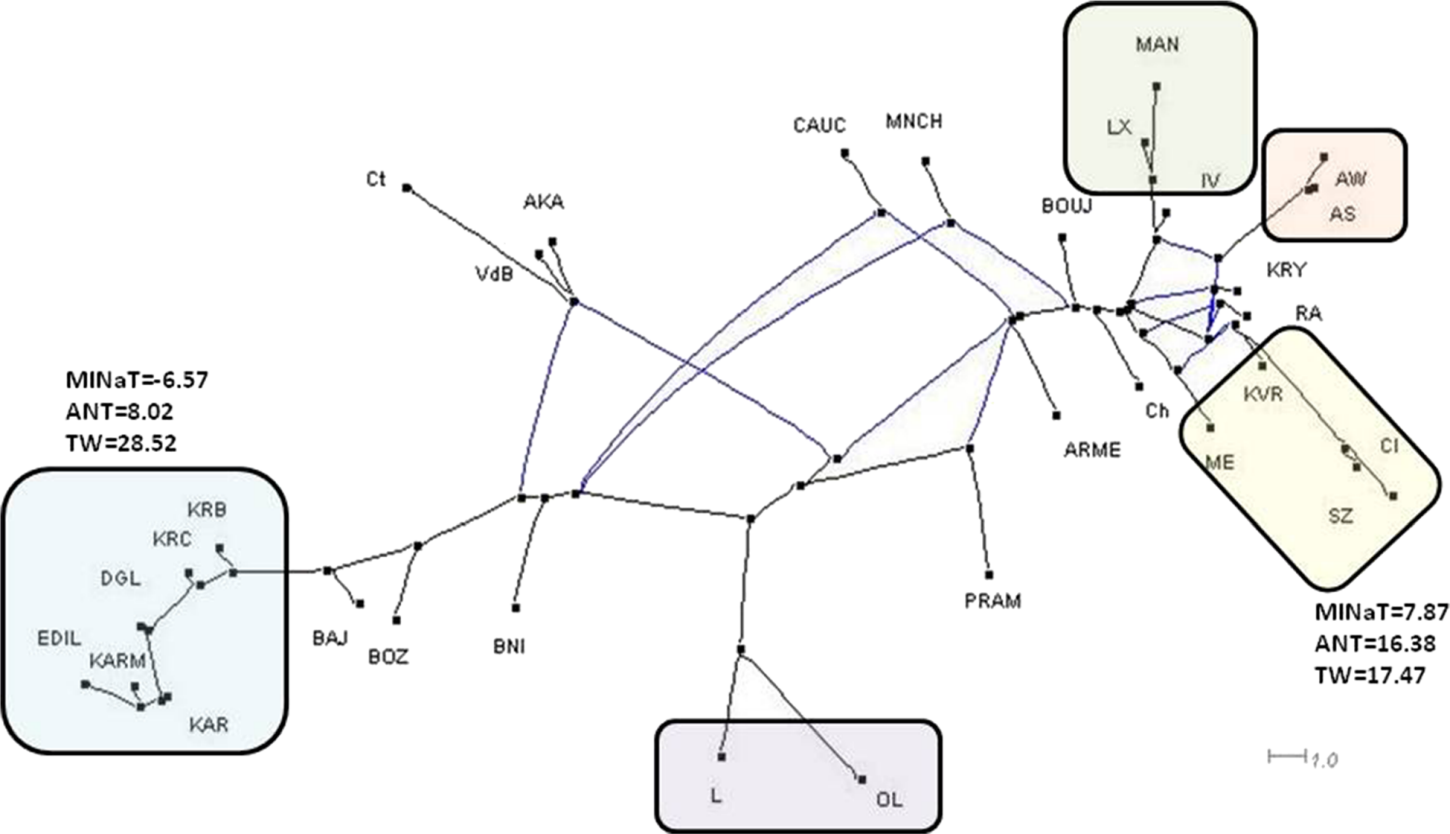
**NeighborNet graph based on Reynold’s distance constructed with the ClusterNetwork splits transformation method for the 31 sheep breeds studied.**

Figure 1 shows NeighborNet graph based on Reynold’s distance constructed with the ClusterNetwork splits transformation method for the 31 sheep breeds studied. The LSFit (which is the least squares fit between the pairwise distances in the graph and the pairwise distances in the matrix) of the NeighborNet was 92.26.

The group constituted by EDIL, KARM, KAR, DGL, KRC, KRB, BAJ and BOZ breeds is outside the reticulations of the NeigborNet graph, indicating a certain degree of separation of this set from the remaining breeds. All these breeds have in common that belong to regions of West Asia and East Europe with high thermal width (arid and semiarid climates). Average, minimum and maximum distances among these breeds were 0.040, 0.000 and 0.167, respectively. The remaining breeds are included in a complex system of reticulations which indicates the existence of a genetic admixture among them [3]. AS and AW breeds are joined in the same branch, as should be expected due to high genetic linkage (Assaf is a synthetic breed from a cross between Awasi and Milkchaff milk breeds). KVR, SZ, ME and Cl breeds come from the same node. All these breeds belong to Mediterranean regions with low thermal width and semi-damp climates. Average, minimum and maximum distances among these breeds were 0.040, 0.000 and 0.080, respectively.

**Figure 2:**
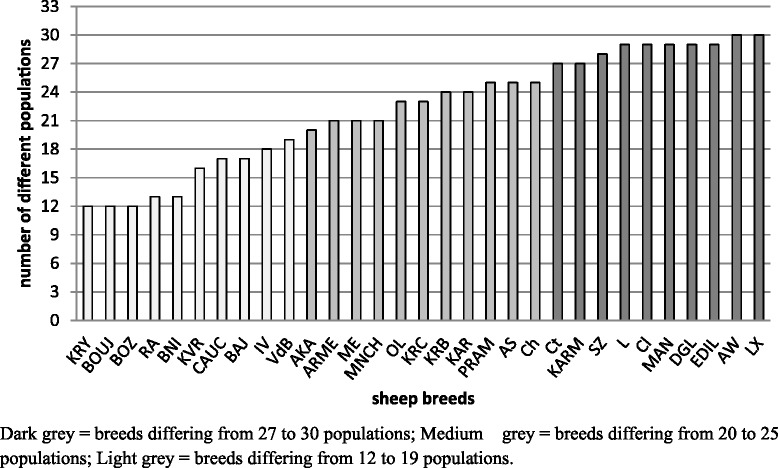
**Histogram of the number of significant different populations (p < 0.05).**

Figure 2 shows the histogram of the number of significant different populations (p < 0.05) for each of the sheep breeds studied using the Exact Test of population differentiation. The number of significant different populations ranged from 12 to 30 and the average was 22.2.

### Tests to detect association of loci frequencies with environmental parameters

#### PLSR

PLSR analysis was conducted including the MAF of six polymorphisms as response variables and 14 environmental variables as predictors (CTY was not included in analyses due to its discrete nature). Polymorphisms considered were g.667_668insC, g.522A > G, g.516_517insG and one polymorphism of each LD block common to most breeds: g.666_667insC, g.660G > C and g.601A > C. For all polymorphisms analyzed, the allele at lower frequency (MAF) was the same in all breeds (I_-668_, I_-667_, A_-601_, A_-522_ and I_-516_). However, the G_-660_ allele of the g.660G > C SNP was the MAF in 25 from the 31 breeds studied.

Basic statistics, and Pearson and Spearman correlations among MAF and environmental variables are shown in Additional file 4 (AF4) and Additional file 5 (AF5), respectively. High negative Pearson (-0.68) and Spearman (-0.70) correlation coefficients were found between MAF of g.667_668insC and g.660G > C (p < 0.0001). A positive and moderate (0.43) Spearman correlation was found for g.667_668insC and g.666_667insC (p < 0.05). Regarding correlations among environmental predictors high (≥0.70) negative correlations (Pearson and Spearman) were found between LAT-MINaT, LAT-ANT, LON-MINaT, MINaT-TW and ANT-TW; and positive between LON-TW, MINaT-ANT and TAR-MxR. Only significant correlations among MAF and environmental predictors were found for g.667_668insC, g.666_667insC and g.660G > C. Similar magnitude but with opposite sign had the correlations found between g.667_668insC and g.660G > C with MINaT, ANT, TW, TAR and MxR.

Table 3 shows Variable Importance in Projection (VIP) and percentage of variance explained by the top two (VT2) PLSR components for each environmental variable. Those variables showing VIP values greater than 0.83 and which VT2 was at least 40%, were retained for posterior analyses. With these criteria, MAXaT, HrMx, HrMi and THI variables were discarded.

**Table 3:** **Variable Importance in Projection (VIP) values and cumulative variance (VT2) explained by the top two factors**

**Variable**	**VIP**	**VT2**
LAT	0.8412	87.389
LON	1.3123	40.001
MINaT	1.0642	94.862
**MAXaT**	**0.7268**	**84.903**
MThm	0.9071	54.214
ANT	0.9467	94.576
TW	1.1786	87.605
TAR	1.0767	76.934
MxR	1.5335	42.358
MiR	1.0205	66.793
HrA	0.8146	72.517
**HrMx**	**0.6934**	**71.635**
**HrMi**	**0.7508**	**67.205**
**THI**	**0.7357**	**82.224**

LAT = latitude; LON = longitude; MAXaT = maximum average temperature; MThm = maximum temperature of the hottest month; MINaT = minimum average temperature; ANT = average annual temperature; TW (MAXaT-MINaT) = thermal width; TAR = total annual rainfall; MxR = maximum rainfall; MiR = minimum rainfall; HrA = relative average annual humidity (%); HrMx = maximum relative humidity (%); HrMi = minimum relative humidity (%); THI = Temperature Humidity Index [22] Variables discarded for posterior analysis are indicated in bold.

A second PLSR analysis including six polymorphisms and ten environmental variables were developed. Predictive Residual Sum of Squares (PRESS) of the complete (14 predictor variables) and reduced (10 predictor variables) models were 0.9726 and 0.9589, respectively, which indicates that the elimination of 4 useless environmental variables improve the prediction model. Reducing the number of predictors, R^2^ (value of explained variation) was also improved from 0.82 (14 predictors) to 0.85 (10 predictors).

**Figure 3:**
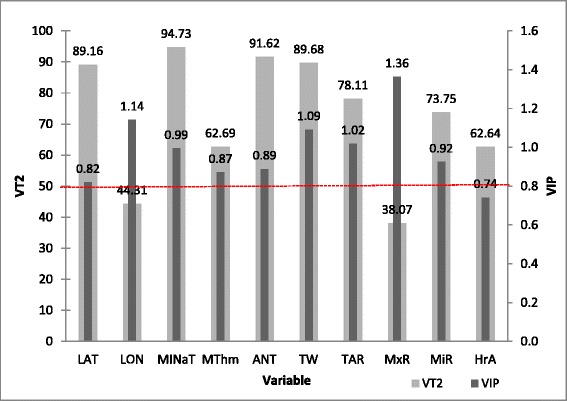
**Variable Importance in Projection values (VIP) and percentage of variance explained by the top two PLSR components (VT2) for each of the ten environmental predictors included in the model.**

Three components were retained using the optimal model determination by the leave-one-out cross validation procedure and the minimum PRESS criteria (van der Voet’s test). The 72.47% of the predictor variation is already explained by just two, but only 24.50% of the response variation is achieved. Figure 3 shows VIP and VT2 values for each of the ten environmental predictors included in the model. Taking into account for both statistics, MINaT, ANT, TW, TAR and MiR were the predictors with the best combination of VIP and VT2. However, environmental variables, as LON and MxR despite having high VIP values showed percentages of the variance explained below 50%.

Predicted variation (Q^2^) values obtained as in equation (1) were calculated for the MAF of the six polymorphisms included in the PLSR model. Q^2^ values were 0.46, 0.47, 0.53, 0.20, 0.10 and 0.33 for I_-668_, I_-667_, G_-660_, A_-601_, A_-522_ and I_-516_, respectively. Only for I_-668_, I_-667_ and G_-660_ Q^2^ values exceed the acceptable threshold (0.4).

**Figure 4:**
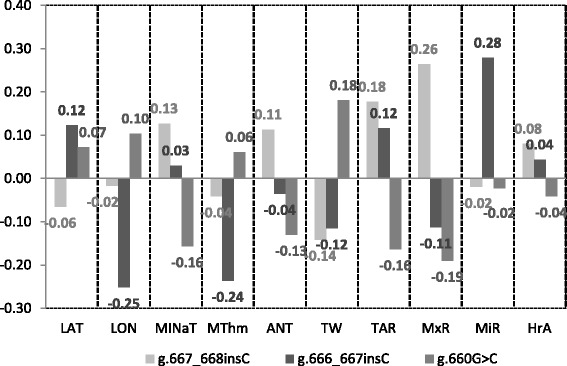
**Regression coefficients for responses (polymorphisms frequencies) with Predicted Variation (Q** ^**2**^ **) values higher than 0.4.**

Regression coefficients for responses with Q^2^ values higher than 0.4, are shown in Figure 4 (Additional file 6 (AF6) showed regression coefficients of scaled and centered variables for all predictors and responses). Absolute values of regression coefficients ranged from 0.02 to 0.28. Interestingly, regression coefficients of I_-668_ and G_-660_ have opposite sign for all environmental predictors, except for MiR, indicating that the MAF at these polymorphisms depends on opposite environmental and geographical circumstances. Thus, the frequency of the I_-668_ and G_-660_ alleles increases and decreases respectively, for higher values of MINaT, ANT, TAR and HrA. Otherwise, high values of LAT, LON and TW are linked to low and high frequencies of the I_-668_ and G_-660_ alleles, respectively.

#### SAM

MATSAM was run for six polymorphisms, g.667_668insC, g.522A > G, g.516_517insG, g.666_667insC, g.660G > C and g.601A > C, and 14 geographic and climatic variables (LAT, LON, MINaT, MAXaT, MThm, ANT, TW, TAR, MxR, MiR, HrA, HrMx, HrMi and THI). Seven alleles at 5 loci were detected as significantly associated with at least one environmental variable with a confidence level of 99.99% (significant threshold ST = 5.952-E05) based on cumulated results from *W* and *G* tests (Table 4). These alleles are involved in 168 significant models according to the *W* and *G* test.

**Table 4:** **Spatial Analysis Method (SAM) cumulated test for molecular sheep data and environmental variables with a significant threshold level of 5.952-E05 (including Bonferroni correction)**

**Marker freq.**	**0.400**	**0.949**	**0.151**	**0.962**	**0.565**	**0.878**	**0.256**	**0.980**	**0.037**	**1.000**	**0.187**	**0.984**
**Marker**	**I**_**-668**_	**D**_**-668**_	**I**_**-667**_	**D**_**-667**_	**G**_**-660**_	**C**_**-660**_	**A**_**-601**_	**C**_**-601**_	**A**_**-522**_	**G**_**-522**_	**I**_**-516**_	**D**_**-516**_
**LAT**	0	0	0	0	**1**	0	**1**	0	0	0	0	0
**LON**	0	0	0	0	**1**	**1**	0	0	0	0	**1**	0
**MINaT**	**1**	0	0	0	**1**	**1**	0	0	0	0	0	0
**MAXaT**	0	0	**1**	0	0	0	**1**	0	0	0	0	0
**MThm**	0	0	**1**	0	0	0	**1**	0	0	0	0	0
**ANT**	**1**	0	0	0	**1**	**1**	0	0	0	0	0	0
**TW**	**1**	0	0	0	**1**	**1**	0	0	0	0	0	0
**TAR**	**1**	0	**1**	0	**1**	0	0	0	0	0	0	0
**MxR**	**1**	**1**	0	0	**1**	0	0	0	0	0	**1**	0
**MiR**	0	0	**1**	0	0	0	0	0	0	0	0	0
**HrA**	0	0	**1**	0	0	0	0	0	0	0	0	0
**HrMx**	0	0	**1**	0	**1**	0	0	0	0	0	0	0
**HrMi**	0	0	**1**	0	0	0	0	0	0	0	0	0
**THI**	0	0	**1**	0	0	0	**1**	0	0	0	0	0

LAT = latitude; LON = longitude; MAXaT = maximum average temperature; MThm = maximum temperature of the hottest month; MINaT = minimum average temperature; ANT = average annual temperature; TW (MAXaT-MINaT) = thermal width; TAR = total annual rainfall; MxR = maximum rainfall; MiR = minimum rainfall; HrA = relative average annual humidity (%); HrMx = maximum relative humidity (%); HrMi = minimum relative humidity (%); THI = Temperature Humidity Index [22].

Cells with ‘1’ indicate that for this model, the null hypothesis is rejected with both the *W* and *G* test.

No association with environmental variables was found for the SNP g.522A > G alleles. MAF alleles I_-668_, I_-667_ and G_-660_ were associated with the highest number of environmental variables, 5, 8 and 8 respectively. The environmental variables related with more number of loci were MxR, ANT, LON, MINaT, TAR and TW (4, 3, 3, 3, 3 and 3, respectively).

**Figure 5:**
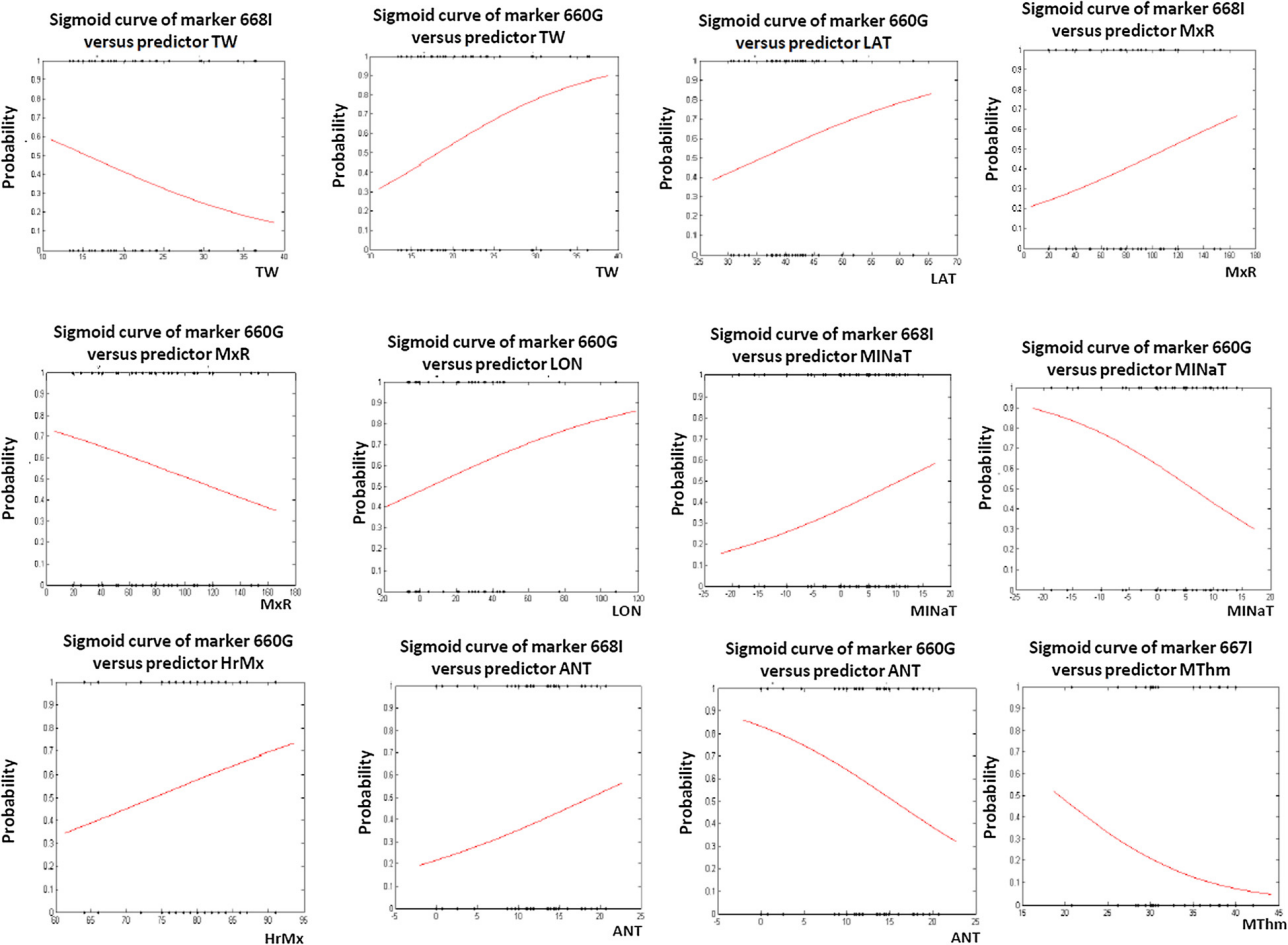
**Correlograms showing polymorphisms alleles significantly associated with environmental variables, which differences in probability of presence of the allele between the extremes of the distribution were higher than 37%.**

Figure 5 shows correlograms of significant associations between markers and environmental variables, which differences in probability of presence of the allele between the extremes of the distribution were higher than 37%. MINaT is the environmental variable for which greatest changes was shown in the probability to find the G_-660_ allele. A decrease in this probability from 0.9 to near 0.3 (60%) was found for G_-660_ when MINaT increases from -22°C to 17°C. An opposite trend was observed for I_-668_. In this case, the likeliness to find de I allele increases from 0.15 to near 0.60 (42.8%) for the same rank of MINaT change. For ANT and MxR the same pattern above described was found. For TW an opposite pattern was observed. So the probability of the G_-660_ allele increases from 0.3 to 0.9 (58%) for 28 units of increment in TW (11 to 38.8), and the probability of the I_-668_ allele decreases from 0.6 to 0.1 (44.1%) for the same rank of TW variation.

### Test to detect loci under selection

#### Bayesian Test of Beaumont and Balding

Table 5 shows expected heterozygosities (H_e_) and *F*_*ST*_ values obtained after 100,000 simulation runs of the LOSITAN and FDIST software using 31 sheep breeds and 6 unlinked polymorphisms at the *HSP90AA1* promoter. Estimated neutral *F*_*ST*_ was 0.072512. Two outlier loci were identified: g.522A > G with a significant (p = 0.02) low *F*_*ST*_ value (0.02) candidate for balancing selection processes, and the g.703_704del(2)A with a significant (p = 0.99) high *F*_*ST*_ value (0.14) candidate for directional selection. With the frequentist approach of FDIST, only sign of balancing selection was found for the g.522A > G SNP.

**Table 5:** **Expected heterozygosities (H**_**e**_ **) and** ***F***_***ST***_ **values obtained after 100,000 simulation runs of the Bayesian Test method of Beaumont and Balding (LOSITAN) and the frequentist method based on moment (FDIST) for six unlinked polymorphisms at the** ***HSP90AA1*** **gene promoter**

	**LOSITAN**				**FDIST**			
**Locus**	**Het**	**Fst**	**P(Simul Fst < sample Fst)**		**Het**	**Fst**	**P(Simul Fst < sample Fst)**	
g.703_704del(2)A	0.48	0.14	0.9957*	directional	0.48	0.14	0.9380	
g.667_668insC	0.34	0.09	0.7658		0.34	0.09	0.4750	
g.666_667insC	0.17	0.07	0.5154		0.17	0.07	0.2534	
g.601A > C	0.25	0.06	0.3443		0.25	0.06	0.1145	
g.522A > G	0.04	0.02	0.0203*	balancing	0.04	0.02	0.0174*	balancing
g.516_517insG	0.16	0.09	0.7049		0.16	0.09	0.4718	

*Indicates significant values.

### *Characterization of the HSP90AA1 promoter in species of the* Caprinae *and* Bovinae *subfamilies*

Aligned sequences of a410 bp amplicon from the *HSP90AA1* gene promoter of a total of 12 species belonging to the Caprinae and Bovinae subfamilies are shown in Additional file 7 (AF7). Species from the *Ovis* genus show 99% similarity with *Ovis aries*, followed by *Capra hircus* and *Ovibos moschatus* both with a similarity of 98%. The least similar species to *Ovis aries* were *Bos mutus* (92%) and *Bos Taurus* (93%). Additional file 8 (AF8) shows haplotypes frequencies in each species studied. In *O. aries* 36 different haplotypes were found. From them only the first four (H1 to H4) had a frequency higher than 10%. In *O. musimon* all the haplotypes found were shared with *O. aries. O. canadiensis* had not polymorphisms in the sequence analyzed. *C. hircus* showed 24 different haplotypes but only one was found in *C. pyrenaica*.

The Tamura 3-parameter model (T92) with evolutionary rates among sites modeled by using a discrete Gamma distribution (+G) with 5 rate categories had the highest fit (lowest BIC value) among the 24 different nucleotide substitution models tested by maximum likelihood. This model was fitted to estimate Evolutionary Divergence between species sequences, to conduct the Tajima’s Neutrality Test and to construct the ML tree.

Table 6 shows estimates of evolutionary divergence over sequence pairs between species. Within the Caprinae subfamily, the *R. pyrenaica* showed the highest percentage of sequence divergence with the remaining species (4.4% with species from the *Ovis* and *Ovibos* genus, 7% with species from the *Capra* genus and 7.6% with *A. lervia*. Interestingly *O. moschatus* was much closer to species of the *Ovis* genus (0.8 to 1%) than to those of *Capra, Ammotragus* and *Rupicapra.* As expected, very low evolutionary divergences among species of the *Ovis* genus and among the species of the *Capra* genus were observed.

**Table 6:** **Estimates of evolutionary divergence between species (below diagonal) and its standard errors (above diagonal)**

	***A. lervia***	***C. hircus***	***C. pyrenaica***	***R. pyrenaica***	***O. moschatus***	***O. aries***	***O. canadiensis***	***O. vignei***	***O. ammon***	***O. musimon***	***B. taurus***	***B. mutus***
*A. lervia*		0.006	0.007	0.014	0.009	0.008	0.008	0.009	0.008	0.008	0.015	0.015
*C. hircus*	0.021		0.003	0.013	0.008	0.007	0.007	0.007	0.007	0.007	0.014	0.014
*C. pyrenaica*	0.022	0.008		0.013	0.008	0.007	0.008	0.008	0.008	0.007	0.014	0.014
*R. pyrenaica*	0.076	0.069	0.070		0.010	0.010	0.010	0.010	0.010	0.010	0.018	0.019
*O. moschatus*	0.036	0.031	0.032	0.045		0.004	0.004	0.004	0.004	0.004	0.014	0.014
*O. aries*	0.031	0.026	0.027	0.046	0.010		0.001	0.002	0.002	0.002	0.013	0.013
*O. canadiensis*	0.028	0.024	0.025	0.043	0.008	0.003		0.002	0.001	0.001	0.013	0.013
*O. vignei*	0.031	0.026	0.027	0.043	0.009	0.005	0.002		0.001	0.002	0.013	0.013
*O. ammon*	0.030	0.025	0.026	0.043	0.008	0.004	0.001	0.001		0.001	0.013	0.013
*O. musimon*	0.029	0.025	0.026	0.044	0.008	0.004	0.001	0.003	0.002		0.013	0.013
*B. taurus*	0.074	0.073	0.073	0.107	0.070	0.065	0.062	0.062	0.062	0.063		0.003
*B. mutus*	0.076	0.075	0.075	0.110	0.073	0.067	0.066	0.066	0.066	0.066	0.006	

Analyses were conducted using the Tamura 3-parameter model (T92 + G).

Table 7 shows polymorphisms detected and its frequencies in the species studied. Within the Caprinae subfamily, the species with more number of polymorphisms, SNPs or INDELs, were *C. hircus* and *O. aries*, with 13 and 11 polymorphic sites, respectively, from which only 6 were shared between them. Also *O. moschatus* and *O. musimon* showed high number of polymorphism, 8 and 7, respectively. The species which shared more number of polymorphisms with *O. aries* (reference species in our work) were *O. musimon* (7), *O moschatus* (7) and *C hircus* (5). In general, within this subfamily, polymorphisms shared among the different species had the same pattern of allele frequency, except for g.528A > G in *O. moschatus* where the A allele showed the highest frequency (0.93) and for g.703_704del(2)A in *R. pyrenaica* where the double A deletion allele was the most frequent (0.75). Exclusive polymorphisms were found in *C. hircus* (8), *A. lervia* (3), *O aries* (2) and *O. moschatus* (1). The two out group species from the *Bovis* genus (*B. mutus and B. taurus*) showed very few polymorphisms and did not share any mutations with the reaming species.

**Table 7:** **Polymorphisms and their frequencies in the wild species analyzed**

**Species**	**g.703_704 del(2)A**		**g.667_668 insC**		**g.666_667 insC**		**g.660G>C**		**g.657A>G**		**g.653C>A**		**g.601A>C**		**g.571G>C**		**g.551A>G**		**g.529G>C**		**g.528A/T>G**		**g.525A>G**		**g.524G>T**		**g.522A>G**		**g.516_517 insG**		**g.498G>C**		**g.482T>C**		**g.468G>T**		**g.463G>A**		**g.456A>G**		**g.444A>G**		**g.406A>G**		**g.395A>G**		**g.384T>G**		**g.320c>G**	
	**-**	**AA**	**C**	**-**	**C**	**-**	**G**	**C**	**G**	**A**	**C**	**A**	**A**	**C**	**G**	**C**	**A**	**G**	**G**	**C**	**A/T**	**G**	**A**	**G**	**G**	**T**	**A**	**G**	**G**	**-**	**G**	**C**	**T**	**C**	**G**	**T**	**G**	**A**	**A**	**G**	**A**	**G**	**A**	**G**	**G**	**A**	**G**	**T**	**C**	**G**
Ovis aries	**0.42**	**0.58**	**0.28**	**0.72**	**0.10**	**0.90**	**0.37**	**0.63**	0	1	0	1	**0.16**	**0.84**	0	1	0	1	0	1	**0.32**	**0.68**	0	1	**0.15**	**0.85**	**0.02**	**0.98**	**0.12**	**0.88**	0	1	0	1	**0.17**	**0.83**	0	1	0	1	**0.15**	**0.85**	0	1	0	1	0	1	0	1
*Ovis ammon*	**0.50**	**0.50**	0	1	0	1	**0.50**	**0.50**	0	1	0	1	0	1	0	1	0	1	0	1	0	1	0	1	0	1	0	1	0	1	0	1	0	1	0	1	0	1	0	1	0	1	0	1	0	1	0	1	0	1
*Ovis canadiensis*	0	1	0	1	0	1	0	1	0	1	0	1	0	1	0	1	0	1	0	1	0	1	0	1	0	1	0	1	0	1	0	1	0	1	0	1	0	1	0	1	0	1	0	1	0	1	0	1	0	1
*Ovis musimon*	**0.53**	**0.47**	**0.47**	**0.53**	**0.05**	**0.95**	**0.47**	**0.53**	0	1	0	1	0	1	0	1	0	1	0	1	**0.19**	**0.81**	0	1	0	1	0	1	**0.13**	**0.87**	0	1	0	1	0	1	0	1	0	1	**0.05**	**0.95**	0	1	0	1	0	1	0	1
*Ovis vignei*	0	1	0	1	0	1	1	0	0	1	0	1	0	1	0	1	0	1	0	1	0	1	0	1	0	1	0	1	0	1	0	1	0	1	0	1	0	1	0	1	0	1	0	1	0	1	0	1	0	1
*Capra hircus*	0	1	**0.18**	**0.82**	**0**	**1**	**0.04**	**0.96**	0	1	1	0	**0.01**	**0.99**	**0.01**	**0.99**	0	1	**0.03**	**0.97**	**0.13**	**0.88**	**0.07**	**0.93**	0	1	0	1	0	1	0	1	0	1	0	1	0	1	**0.05**	**0.95**	**0.01**	**0.99**	**0.23**	**0.77**	**0.16**	**0.84**	**0.64**	**0.36**	**0.08**	**0.92**
*Capra pyrenaica*	0	1	0	1	0	1	0	1	0	1	1	0	0	1	0	1	0	1	0	1	0	1	0	1	0	1	**0.13**	**0.87**	0	1	0	1	0	1	0	1	0	1	0	1	0	1	1	0	0	1	0	1	0	1
*Ovibos moschatus*	**0.14**	**0.86**	**0.43**	**0.57**	**0.40**	**0.60**	**0.10**	**0.90**	0	1	1	0	0	1	0	1	**0.30**	**0.70**	0	1	**0.93**	**0.07**	0	1	0	1	**0.03**	**0.97**	0	1	0	1	0	1	0	1	0	1	0	1	**0.47**	**0.53**	0	1	0	1	0	1	0	1
*Rupicapra pyrenaica*	**0.75**	**0.25**	0	1	0	1	**0.25**	**0.75**	0	1	0	1	0	1	0	1	0	1	0	1	0	1	0	1	0	1	0	1	0	1	0	1	0	1	0	1	0	1	0	1	0	1	0	1	0	1	0	1	0	1
*Ammotragus lervia*	0	1	0	1	0	1	0	1	**0.82**	**0.18**	**0.04**	**0.96**	0	1	0	1	0	1	0	1	0	1	0	1	0	1	0	1	0	1	**0.18**	**0.82**	0	1	0	1	0	1	0	1	0	1	0	1	0	1	0	1	0	1
*Bos mutus*	0	1	0	1	0	1	-	-	0	1	1	0	0	1	0	1	0	1	0	1	0	1	0	1	0	1	**T**	**T**	0	1	0	1	**0.92**	**0.08**	0	1	0	1	0	1	0	1	0	1	0	1	0	1	0	1
*Bos taurus*	0	1	0	1	0	1	-	-	0	1	1	0	0	1	0	1	0	1	0	1	**0.15**	**0.85**	0	1	0	1	**T**	**T**	0	1	0	1	0	1	0	1	**0.85**	**0.15**	0	1	0	1	0	1	0	1	0	1	0	1

In bold are polymorphic positions.

The three INDELs g.703_704del(2)A, g.667_668insC and g.666_667insC existed simultaneously only in *O. moschatus*, *O. musimon* and *O. aries. C hircus* had the two contiguous g.667_668insC and g.666_667insC, and *O. ammon* and *R pyrenaica* the g.703_704del(2)A. The highest frequency of the I_-668_ allele, related with heat stress tolerance, was found in *O. musimon* (0.47) and *O. moschatus* (0.43) followed by *O. aries* (0.28) and *C. hircus* (0.18). The SNP g.660G > C seems to be exclusive of the Caprinae subfamily. Unfortunately, this region is a sequence of several consecutive cytosines and therefore is difficult to know if there is not mutation at -660 position or if the C_-660_ allele is fixed in *Bos*. Anyway, C appears to be the wild allele of the g.660G > C SNP.

Tajima’s Neutrality Test [23] conducted for the sequences of the *HSP90AA1* promoter was -2.56 (p_value < 0.05 [24]) which reveals an excess of low frequency polymorphisms relative to expectation (D_L_ = -1.78). This fact could indicate a purifying selection removing alleles that diminish animal’s biological fitness but also the presence of “young” beneficial mutations going to higher frequencies.

**Figure 6:**
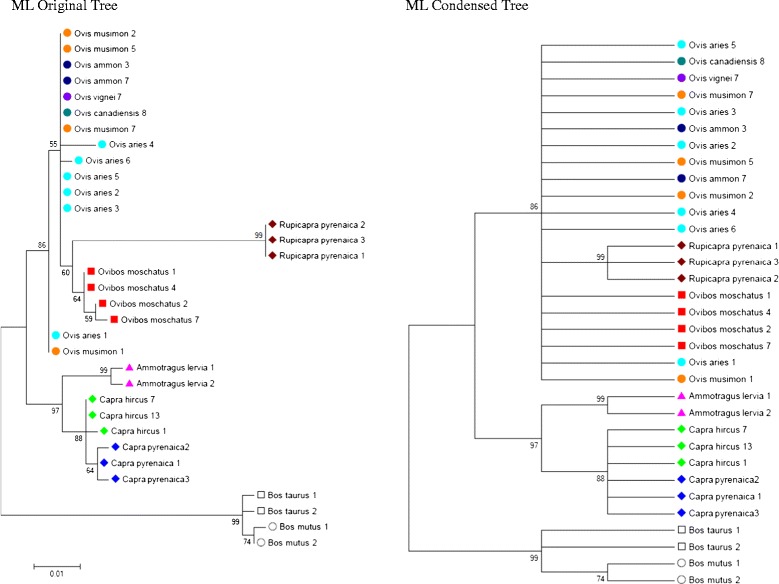
**Molecular Phylogenetic analysis by Maximum Likelihood method developed with MEGA6.** Original and condensed ML trees.

Figure 6 shows Maximum Likelihood bootstrap original and condensed trees based on the Tamura 3-parameter model and inferred from 5000 replicates. The tree was constructed considering only haplotypes with frequencies higher than 0.05. Branches corresponding to partitions reproduced in less than 50% bootstrap replicates are collapsed. The analysis involved 33 nucleotide sequences. There were a total of 410 positions in the final dataset. Out-group species (*B. taurus* and *B.mutus*) were located in a separate branch with a high bootstrap percentage (99). One branch were constituted by species of the *Capra* and *Ammotragus* genus (97) and other branch by those of the *Ovis*, *Rupicapra* and *Ovibos* ones (86). In the ML consensus tree, *O. moschatus* was located as a sister species of *R. pyrenaica*. Species from the *Ovis* genus (*O. aries; O. musimon, O. vignei, O. ammon* and *O. canadiensis*) appear mixed since many haplotypes are shared among them and promoter sequences showed a high degree of similarity.

## Discussion

Previous studies from our group pointed out the existence of different expression profiles in sheep carrying alternative genotypes of some polymorphisms located at the *HSP90AA1* gene promoter depending on environmental temperatures [18-20]. The join genotype of the g.667_668insC and the g.660G > C polymorphisms had the highest effect on the expression rate of the gene and on the sperm DNA fragmentation levels [20,21]. Animals carrying the II_-668_-CC_-660_ genotype showed higher expression rate (Fold Change = 3.1 to 3.5) of the *HSP90AA1* gene than those with DD_-668_-CC_-660_, DD_-668_-CG_-660_ and DD_-668_-GG_-660_ under heat stress environmental conditions (27°C average daily temperature and 34°C maximum daily temperature) [19,20]. At the phenotypic level the II_-668_-CC_-660_ combined genotype had the lowest values of sperm DNA fragmentation (up to 3.5 times less) compared with the remaining genotypes, when heat stress events occurs along the spermatogenesis process [20,21].

The results above described may contribute to clarify the phylogeographic relationship for some sheep breeds and the opposite correlations observed between the frequency of the I_-668_ and G_-660_ alleles and some climatic and geographic variables from the locations where they are reared. Since the I_-668_ allele is responsible of the upregulation of the gene under heat stress conditions, high frequency of this allele is expected to be found in climates with high minimum (MINaT) and average annual (ANT) temperatures (positive regression coefficients, 0.11 and 0.13) and therefore with low thermal width (TW) (negative regression coefficient, -0.14). Despite no significant correlation was found among Total Annual Rainfall (TAR) and Maximum Rainfall (MxR) with MINaT and ANT, the frequency of the I_-668_ allele seems to be also associated with changes in these last variables. Thus the I_-668_ allele frequency is high in climates with high MINaT, ANT, TAR and MxR values and low TW values.

Looking at climatic variables of countries where those breeds are reared (Table 8) we can observe that Semi Arid (SA) regions showed greater average TW (24.87) and average ANT (13.70) and lower average MINaT (-1.15) than Semi Damp (SD) locations (average TW = 18.69, ANT = 11.04 and MINaT = 4.47). Also in SD locations TAR and MxR values (626 and 103, respectively) are much higher than those of SA regions (372 and 58, respectively). Therefore, as SD are heater than SA regions, it is possible to hypothesize that heat events accompanied with high rainfall in SD regions could be more stressful since thermal stress increases when high temperatures and high relative air humidity go together [25]. In this sense, Paim and colleagues [26] described that THI (an index that combines air temperature and relative air humidity) had a great influence on animal superficial temperatures, demonstrating that this is able to characterize the animal response to environment. However in this work any association was found between this variable and polymorphisms frequencies.

**Table 8:** **Sheep breeds, locations, countries and continents of origin and climatic and geographic variables**

**Breed**	**ID breed**	**Location**	**Country**	**Continent**	**N**	**Lat**	**LON**	**MINaT**	**MAXaT**	**MThm**	**ANT**	**TW**	**TAR**	**MxR**	**MiR**	**HrA**	**HrMx**	**HrMi**	**THI**	**CTY**
Akkaraman	AKA	Konya	Turkey	Asia	23	37.60	32.30	-0.20	23.10	30.10	11.50	23.30	315	41	4	60	80	40	22.02	SA
Kazakh Arkhar-Merino	ARME	Alma Ata	Kazakhstan	Asia	18	43.45	77.04	-6.10	23.60	29.40	9.10	29.70	653	107	26	62	77	45	22.52	SD
Assaf	AS	Haifa	Israel	Asia	30	31.86	35.21	14.00	29.90	40.00	20.70	13.40	534	148	0	68	72	60	28.36	SD
Awassi	AW	Haifa	Israel	Asia	30	31.86	35.21	14.00	27.40	40.00	20.70	13.40	534	148	0	68	72	60	26.11	SD
Bajdarak	BAJ	Irkutsk	Russia	Asia	22	51.83	107.43	-18.80	17.50	34.00	0.00	36.30	468	120	9	75	86	58	17.26	SA
Bni Guil	BNI	Fez	Morocco	Africa	27	33.56	4.59	9.60	27.10	35.80	17.80	17.50	537	85	1	67	79	56	25.80	SD
Boujaad	BOUJ	Marrakech	Morocco	Africa	24	32.86	-6.96	12.20	28.30	39.00	19.60	16.10	282	41	1	58	66	47	26.49	SA
Bozakh	BOZ	Kirovabad	Azerbaijan	Asia	24	40.53	46.02	2.70	25.10	30.90	13.90	22.40	294	51	11	65	78	53	23.94	SA
Caucasian	CAUC	Astrakan	Russia	Asia	25	45.71	42.88	-5.50	25.20	30.60	10.00	30.70	216	25	10	70	86	54	24.20	A
Churra	Ch	León	Spain	Europe	23	42.58	-5.65	3.10	19.60	37.00	10.90	16.50	555	70	24	68	83	55	19.08	SD
Churra Lebrijana	Cl	Jerez	Spain	Europe	26	36.75	-6.06	10.70	25.70	38.00	17.70	15.00	587	109	2	67	79	54	24.54	SD
Churra Tensina	Ct	Huesca	Spain	Europe	33	42.08	-0.33	4.90	23.40	37.00	13.60	18.50	534	62	20	63	81	48	22.37	SD
Daglic	DGL	Eskisehir	Turkey	Asia	24	38.50	30.30	-0.10	21.40	28.60	11.10	21.50	388	51	9	70	84	59	20.75	SA
Kazakh Edilbai	EDIL	Aktjubinsk	Kazakhstan	Asia	30	52.32	77.03	-14.10	22.40	39.00	4.70	36.50	311	33	19	66	82	50	21.56	SA
Ivesi	IV	Diyarbakir	Turkey	Asia	15	37.50	40.10	1.50	31.00	38.10	14.90	29.50	498	76	1	55	77	29	28.68	SA
Russian Karakul	KAR	Astradan	Russia	Asia	15	45.37	46.04	-5.50	25.20	42.00	10.00	30.70	216	25	10	70	86	54	24.20	A
Moldavian Karakul	KARM	Kisinev	Moldavia	Europe	15	46.93	28.75	-3.30	20.90	37.00	9.60	24.20	547	75	27	73	86	62	20.36	SD
Karabakh	KRB	Kirovabad	Azerbaijan	Asia	24	39.82	46.70	-0.40	23.60	38.00	11.90	24.00	294	51	11	65	78	53	22.60	SA
Karachai	KRC	Krasnojarsk	Azerbaijan	Asia	28	43.04	44.21	-16.00	18.20	37.00	0.80	34.20	450	79	12	68	76	53	17.82	SA
Karayaka	KRY	Samsun	Turkey	Asia	22	41.20	36.20	6.90	22.70	26.20	14.30	15.80	692	89	29	72	79	65	21.98	SD
Kivircik	KVR	Bursa	Turkey	Asia	16	40.10	29.00	5.20	24.10	30.30	14.40	18.90	706	118	16	72	78	62	23.26	SD
Finnsheep	L	Jyvaskyla	Finland	Europe	30	62.31	27.17	-10.00	15.70	20.80	2.60	25.70	640	91	30	80	91	65	15.62	SD
Latxa	LX	Vitoria	Spain	Europe	41	42.88	-2.73	4.70	19.10	30.00	11.40	14.40	782	89	42	75	84	71	18.74	SD
D’Man	MAN	Ouarzazate	Morocco	Africa	26	30.93	-6.90	9.30	29.50	40.00	18.90	20.20	110	19	1	41	64	22	26.74	A
Spanish Merino	ME	Cáceres	Spain	Europe	29	39.29	-6.22	8.20	25.60	39.00	16.00	17.40	488	65	4	57	76	35	24.11	SA
Manchega	MNCH	Albacete	Spain	Europe	60	38.95	-1.85	5.00	24.10	38.00	13.50	19.10	367	52	9	63	79	44	22.99	SA
Olkuska	OL	Cracovia	Poland	Europe	30	49.78	21.34	-2.90	19.30	30.00	8.60	22.20	679	95	33	80	87	72	19.00	SD
Pramenka	PRAM	Belgrado	Servia	Europe	29	44.74	20.44	0.50	21.70	28.30	11.80	21.20	694	95	42	69	82	60	21.00	SD
Rasa Aragonesa	RA	Zaragoza	Spain	Europe	42	41.66	-1.01	6.20	24.30	35.00	14.60	18.10	314	38	15	65	77	53	23.23	SA
Sakiz	SZ	Izmir	Chios	Asia	26	38.30	27.10	8.60	22.50	32.70	17.70	18.90	693	153	4	62	72	49	21.55	SD
Valle del Belice	VdB	Palermo	Italy	Europe	29	37.69	13.02	11.60	25.40	30.20	18.20	13.80	654	106	5	69	75	63	24.34	SD

N: number of animals; LAT = latitude; LON = longitude; MAXaT = maximum average temperature; MThm = maximum temperature of the hottest month; MINaT = minimum average temperature; ANT = average annual temperature; TW (MAXaT-MINaT) = thermal width; TAR = total annual rainfall;; MxR = maximum rainfall; MiR = minimum rainfall; HrA = relative average annual humidity (%);HrMx = maximum relative humidity (%);HrMi = minimum relative humidity (%);THI = Temperature Humidity Index THI = T°C – (0.31-0.31RH)x(T°C-14.4) [22], T = temperature in °C, RH = relative humidity in %/100. THI < 22.2 = absence heat stress; 22.2 > THI < 23.3 = moderate heat stress; 23.3 > THI < 25.6 = severe heat stress; 25.6 > THI = extreme severe heat stress; CTY = climate type (arid A = 0-250 mm; semi arid SA = 250-500 mm; semi damp SD = 500-1000 mm; damp D = 1000-2000 mm; very damp VD= > 2000 mm).

Regarding the G_-660_ allele in the gene promoter, opposite results than those of the I_-668_ were observed, which agree with the transcription results above mentioned. The G_-660_ allele is linked to the lowest expression rates of the *HSP90AA1* gene under both heat stress and mild temperature conditions. Therefore high frequencies of such allele are only expected in breeds reared in regions with low MINaT and ANT temperatures and high TW, in which heat is not a critical source of stress. The negative association of the G_-660_ frequency with MINaT (-0.16) and ANT (-0.13) and positive with TW (0.18) agree with such expectations. However, high frequencies of the C_-660_ allele were found in all kind of locations but predominating in breeds reared in hot climates. This could be due to the genetic exchange that occurred during the development of modern breeds more than to adaptation processes. Linkage disequilibrium (LD) between g.667_668insC and g.660G > C is little than 0.20 in the whole breeds and range between 0.001 and 0.540 across breeds, however D_-668_ and G_-660_ alleles are completely linked in the 836 animals genotyped, constituting the most thermo sensible haplotype [20].

On the basis of Reynold’s distances, two groups of breeds showing the minimum distances between breeds within group and maximum distances with breeds of the other group can be established. The first group was constituted by KRC, KRB, KAR, DGL, KARM and EDIL breeds and the second group by ME, PRAM, SZ, AS, KVR and Cl breeds. Among breeds of these two groups, average, minimum and maximum distances were 0.267, 0.064 and 0.604, respectively. In these two groups of breeds, opposite frequencies of the two polymorphism most related with gene expression differences (g.667_668insC and g.660G > C) were observed. Thus, in the first group of breeds the average frequencies of the I_-668_ and G_-660_ alleles were 0.08 and 0.61, respectively. In all these breeds the G_-660_ allele was that with the maximum frequency and the I_-668_ allele had a frequency <13%. In the second group of breeds average frequencies of I_-668_ and G_-660_ alleles were 0.41 and 0.23, respectively. In all these breeds the I_-668_ allele frequency was >30% and the G_-660_ allele had a frequency <33%. Interestingly, all breeds from group 1, except KARM, are reared in SA or A climates, mainly from Asian regions. On the contrary, all breeds from group 2, except ME, are reared in SD Mediterranean climates. Average MINaT, ANT, TW, TAR and MxR were -6.57, 8.02, 28.52, 367.63 and 52.30, respectively, in group 1 and 7.87, 16.38, 17.47, 617 and 114.67, respectively, in group 2. Q^2^ values higher than 0.4 were only found for I_-668_, I_-667_ and G_-660_ suggesting the action of natural selection in driving the differential allele frequency distribution of these polymorphisms among sheep populations. Therefore, a correlation between genetic (allele frequencies) and environmental (climatic parameters) variables among some sheep breeds have been established which demonstrates that despite of the great admixture existing among them and its domestication status, some footprints of the natural selection action can be glimpsed. This fact may be due to the general low artificial selection exerted over breeds of this species and their semi-extensive or extensive management conditions which may have retained some genes related with adaptation to environmental conditions existing in nature. Thus, breeds reared in SD climates, in which high temperatures and humidity are sources of physiological stress, have high frequency of alleles (I_-668_ and C_-660_) related to higher expression rates of the *HSP90AA1* gene as response to heat stress. However, low frequencies of these alleles were only found in those breeds reared in climates in which heat and humidity levels are not enough to induce a heat stress response. The frequencies of A_-601_, A_-522_ and I_-516_ alleles (Q^2^ values < 0.4) are not influenced by climatic conditions and therefore its presence in the *HS90AA1* gene promoter seem to have no impact in the adaptation to environment of the ovine species. This finding was already suggested by [19] by notice that these polymorphisms did not produce expression differences among genotypes when comparing RNA samples obtained under heat stress and thermo-neutral conditions.

In a large study where 49,034 SNPs were genotyped in 74 sheep breeds, [3] some signs of directional selection in two candidate genes located at chromosome 18 (*F*_*ST*_ = 0.428), in which also the *HSP90AA1* gene is located, were found. One of them was *ABHD2* (abhydrolase domain containing 2) which has, among other functions, a role in the response to wounding. This protein that interacts with UBC (polyubiquitin C) has a high expression rate in testicle (BioGPS. biogps.org) and correlates with *HSPA1L* (Heat shock protein 70 kD like). Hsp70 is a well known protein involved in the heat shock response which is part of the Hsp90 complex. Therefore, although authors [3] recognize that the identification of adaptive alleles has not been achieved, some footprints of directional selection over genes more or less directly related with adaptive traits can be found.

When assessing evidence for an ecocline, it is crucial to control for population history and structure, for accurately assessing whether a correlation between a genetic variant and geographic or climate variables is due to natural selection [27]. For example, if migration patterns correspond closely with variation in a particular climate variable, the correlations between neutral alleles and that climate variable may be high even if selection has not acted on the locus. Conversely, if selection effects are lower to that of population structure on allele frequencies, correlations may be underestimated if population history is not taken into account [4].This is the reason why PLSR and SAM approaches cannot be used independently, without comparing results with specialized statistic methods based on population genetics theories, and focus on the analysis of genetic data as the Bayesian Test of Beaumont and Balding (LOSITAN). Thus, among all loci-environment associations detected by PLSR and SAM methods, only the frequency of two polymorphisms, the g.703_704del(2)A and the g.522A > G, seems to be under the action of some selective process. The g.703_704del(2)A showed a high *F*_*ST*_ outlier which makes it a candidate to directional selective processes. The low *F*_*ST*_ outlier of the g.522A > G SNP reveals the possibility of balancing selection acting over its frequency. The g.703_704del(2)A is highly linked with the g.660G > C SNP (r^2^ = 0.86 in the whole data) ranging r^2^ values in most breeds from 0.84 to 1. Thus, directional selection predicted for the g.703_704del(2)A could be extended to the SNP g.660G > C for which differential expression of the *HSP90AA1* gene has been assessed depending on genotype [19,20], but not with the g.667_668insC. The high degree of conservation in LD phase found in this sort sequence in almost breeds, independently of their geographic origin, could indicate that high levels of gene flow have occurred between populations following domestication, as is suggested by Kijas and coworkers [3], but also, a selection pressure exerted over this DNA region [28].

The *Bovidae* family includes more species than any other extant family of large mammals, but their phylogenetic relationships remain largely unresolved in part because it appears to represent a rapid, early radiation into many forms without clear connections among them [29]. Furthermore, certain morphological traits have evolved several times within the family to create evolutive convergences that obscures true relationships [30]. The subfamily Caprinae includes bovids adapted to extreme climates and difficult terrains. Fossil records are poorly documented but the group first appeared during the upper Miocene [31]. In a recent work, a complete estimate of the phylogenetic relationships in Ruminantia has been proposed combining morphological, ethological and molecular information [29]. The resolution of the supertree varies among groups and some component clades, particularly Caprinae (67.7%), are much less well resolved than others (e.g. Bovinae*,* 95.7%). In particular, the position of the genera *Budorcas* and *Ovibos* has been controversial, having at times constituted the tribe Ovibovini, and at others been separated and located in different tribes. In general, the genus *Ovis* is split into a “New World” clade represented by *O. dalli* and *O. canadensis* and an “Old World” clade including the two sister species *O. vignei* and *O. aries,* on the one hand, and *O. ammon,* on the other hand [29,32]. In our work, haplotypes from *O. vignei, O canadiensis* and *O. musimon* appeared mixed with those from *O. aries. O. aries* and *O. musimon* share many polymorphic sites (7) as expected from the past hybridization between both species.

Ropiquet and Hassanin [33] using mitochondrial and nuclear DNA sequences located *A. lervia* closer to goats (*Capra*) and *O. moschatus* closer to *R. pyrenaica*. However, in recent works [34,35] *A. lervia* was closer to *Rupicapra* genus within the Caprina tribe and *O. moschatus* was distant from them within the Ovibovina tribe. Our tree located *A. lervia* as a sister species of *C. hircus* and *C. pyrenaica* (boosttrap proportion = 97) and *R. pyrenaica* closer to *O moschatus* (bootstrap proportion = 60). In the work of Matthee and Davis [36] using data from nuclear DNA a politomy for *C. hircus, O. moschatus* and *O. aries* was found. However, when analyzing nuclear DNA joined to mtDNA data, *C. hircus* and *O. aries* appear as sister species separated from *O. moschatus*. In our work we have observed a relative high similarity between *O. moschatus*, *O. aries and O. musimon* species regarding polymorphism shared among them.

Although *O. moschatus* is currently restricted to Greenland and the Arctic Archipelago [37], a higher frequency of alleles related with the heat stress response (I_-668_ = 0.43; C_-660_ = 0.90) were found in this species. Fossils of this species have occasionally found in southwest Europe, so that’s why it seems that *Ovibos* did not inhabit exclusively cold tundra during the Pleistocene [37]. *Praeovibos,* an older morphotype of *O moschatus*, does not appear to have been restricted to inhabiting cold climates as its remains have also been identified in temperate and Mediterranean forest [38,39]. In contrast to modern *Ovibos, Praeovibos* was distributed over much more southern latitudes, samples have been found as far south as France and Spain [38-40], which indicates that *Praeovibos* is an early more cosmopolitan form of muskox [37]. Could these high frequencies of alleles related with the heat stress response found in *O moschatus* came from its *Praeovibos* ancestor? Lent [41] indicates that muskox is sensitive to both climate warming and fluctuations, that is why Campos and colleagues [37] hold these factors responsible of the actual confinement of the muskox to Greenland and the Arctic Archipelago but not a human impact. Our results regarding the polymorphisms of the *HSP90AA1* gene in this species seems to indicate that the actual muskox is genetically well prepared to tolerate warm climates. Therefore, which could be the reasons to its actual geographic limitations? Climate change is known to affect not only animal’s thermo sensitivity but also by triggering vegetation change [39,42]. Increasing temperature pushed the adaptive vegetation balance firmly towards bogs, shrub tundra, forest and low-nutrient acidic soils, which resulted in communities of conservative plants highly defended against herbivore and supporting a small biomass of large mammals [43]. Palmqvist and coworkers [44] in an ecomorphological analysis of the early Pleistocene fauna of Venta Micena (Orce, Guadix-Baza basin, SE Spain), provide interesting clues on the physiology, dietary regimes, habitat preferences and ecological interactions of large mammals.

Unexpectedly, *A. lervia* which colonizes arid and hot areas of the rocky mountains of north Africa (Sahara and Magreb) is not polymorphic for the mutation most associated to the upregulation of the *HSP90AA1* gene induced by heat stress events. It is probably that in this species, as in the *Bos* genus, other genetic mechanisms exist to cope with stress imposed by climatic conditions.

Regarding those polymorphisms for which our group has detected some relation with hot climates adaptation by its association with the expression rate of the *HSP90AA1* gene under heat stress conditions (g-.667_668insC and g.660G > C), it’s noteworthy that they were only segregating in *C. hircus, O. moschatus, O.musimon* and *O. aries*. Because there were only one sample of *O. vignei* and two of *O ammon*, any conclusion from these two species can be extracted. It seems reasonable to hypothesize that these polymorphisms could come from an ancestral species common to the *Ovis, Ovibos* and *Capra* genera but not to *Ammotragus.* However also it is possible that the evolvability of this gene may be due to its physical susceptibility to mutagenesis and therefore that the similitudes/differences found in the species analyzed does not be related with their phylogenetic relations.

## Conclusion

We have assessed that despite the domestication process occurred 11,000 years BP, sheep breeds showed some genetic footprints related to climatic variables existing in the regions where they are reared. Thus artificial selection carried out by humans to improve productive traits in this species seems to be occurred concurrently with natural selective forces for traits related with the adaptation to environmental conditions. Adaptation of breeds to heat climates can suppose a selective advantage to cope with global warming caused by climatic change. Polymorphisms of the *HSP90AA1* gene detected in the *Ovis aries* species can be used in selection programs to improve animals resistance to heat environments. Mutations of the ovine *HSP90AA1* gene promoter are also been found in wild species from the Caprinae subfamily, indicating a great antiquity of these mutations which can help us to elucidate how climatic conditions have evolved in the past.

## Methods

### Ethics statement

The current study was carried out under a Project License from the INIA Scientific Ethic Committee. Animal manipulations were performed according to the Spanish Policy for Animal Protection RD 53/2013, which meets the European Union Directive 86/609 about the protection of animals used in experimentation. We hereby confirm that the INIA Scientific Ethic Committee (IACUC) has approved this study.

### Animal material, nucleic acid isolation, DNA amplification and SNPs genotyping

Animals from 31 sheep breeds from Europe, Asia and Africa and from 11 species of the Caprinae (9) and the Bovinae (2) subfamilies constitute the biological material of this work. Tables 8 and 9 shows breeds, species, number of animals from each breed and species, location, country, continent and climatic and geographic variables. Additional file 9 (AF9) contains the genotypes of all animals analyzed for each polimorphisms existent in each species.

**Table 9:** **Wild species from the Caprinae and Bovinae subfamilies**

**Subfamily**	**Genus**	**Species**	**Name**	**N**	**Breeds**	**Country**	**Continent**
Caprinae	*Ovibos*	*Ovibos moschatus*	Muskox	15		Norway	Europe
	*Capra*	*Capra hircus*	Domestic goat	57	7 breeds	Spain	Europe
		*Capra pyrenaica*	Iberian Ibex	5		Spain	Europe
	*Ammotragus*	*Ammotragus lervia*	Barbary sheep	14		Morocco	Africa
	*Ovis*	*Ovis aries*	Sheep	836	31 breeds	-	-
		*Ovis ammon*	Argali	1		Mongolia	Asia
		*Ovis canadiensis*	Big Horn	8		Canada	North America
		*Ovis vignei*	Urial	1		Afganistan	Asia
		*Ovis orientalis musimon*	European Muflon	31		Corsica	Europe
	*Rupicapra*	*Rupicapra pyrenaica*	Pyrenean chamois	2		Spain	Europe
Bovinae	*Bos*	*Bos taurus*	Cattle	43	5 breeds	Spain	Europe
		*Bos mutus*	Yak	13		Tibet	Asia

Goat breeds: Guadarrama, Girgentana, Maltese, Angora, Blanca Celtibérica, cross.

Cattle breeds: Holstein, Avileña, Serrana, Pirenaica, Parda de Montaña.

Sheep breeds: those used in this work.

Peripheral whole blood samples were collected in order to analyse 11 polymorphisms of interest located at the *HSP90AA1* promoter [20]. Polymorphisms genotyped were: g.703_704del(2)A; g.667_668insC (rs397514115); g.666_667insC; g.660G > C (rs397514116); g.601A > C (rs397514117); g.528A > G (rs397514269); g.524G > T (rs397514270); g.522A > G (rs397514271); g.516_517insG (rs397514268); g.468G > T (rs397514272); g.444A > G (rs397514273). *HSP90AA1* promoter sequencing was done in all animals according to Salces-Ortiz and coworkers [19].

### Polymorphisms characterization and linkage disequilibrium estimation

PLINK software [45] was used to estimate linkage disequilibrium among all pairs of the 11 SNPs measured as r^2^ in the whole sheep data and in each breed separately. Hardy-Weinberg equilibrium exact test, observed and expected heterozygosis for each breed were also calculated using PLINK.

### Phylogenetic relationship between sheep breeds

The relationship between breeds was examined using the Reynold’s distance metric [46]. Reynold’s distance (D = -ln(1-F_ST_) matrix was estimated performing 90,000 permutations and a significance level of 0.05 was established. An Exact Test of population differentiation with a significance level of 0.05 was performed to test the hypothesis of a random distribution of individuals between pairs of populations [47,48], running 100,000 Markov chain and 10,000 dememorization steps. The histogram of the number of populations which are significantly different (p < 0.05) from a given population was generated. All analyses were made by using the ARLEQUIN 3.1 software [49].

A NeighborNet graph was constructed from the matrix of Reynold’s distances using SplitsTree4 V4.13.1 software [50].

### Tests to detect association of loci frequencies with environmental parameters

#### Partial least square regression (PLSR)

Partial Least Squares multiple regression (PLSR) was applied to model the relationships between polymorphisms allele frequencies found in the 31 sheep breeds genotyped and a matrix describing environmental factors (14 geographical and climatic variables) as in Fumagalli et al. [51]. The specific algorithm used to compute extracted PLSR factors was SVD (Singular Value Decomposition). SVD is a factorization of a matrix which bases the extraction on the singular value decomposition of *X’Y.*

For each polymorphism the relationship between population allele frequency matrix (F) of dimension 31x1 and environmental predictors matrix (M) of 31x14 dimensions was assessed. F describes minor allele frequency (MAF) at each breed for the examined polymorphism, whereas M describes all the 14 environmental variables for each population.

In order to evaluate the fit of a model, values of explained variation, R^2^, and predicted variation, Q^2^, were computed as in [51]. Q^2^ provides a measure of how well a model predicts the observed data using a cross-validation procedure, which is in this case how well a model of environmental variables predicts the observed distribution of allele frequencies among breeds. If allele frequencies covary with environmental variables Q^2^ will be large. Acceptable values of R^2^ and Q^2^ are totally dependent on the nature of the data. Lundstedt et al. [52] propose Q^2^ > 0.4 and R^2^ > 0.7 as acceptable thresholds for biological data.

The number of factors chosen is usually the one that minimizes the Predictive Residual Sum of Squares (PRESS). However, often models with fewer factors have PRESS statistics that are only marginally larger than the absolute minimum. To address this, van der Voet [53] proposed a statistical test for comparing the predicted residuals from different models. By applying the van der Voet’s test, the number of factors chosen is the fewest with residuals that are insignificantly larger than the residuals of the model with minimum PRESS.

Uninformative variable elimination to remove those variables that are useless was made in the basis of two filter criteria: the Variable Importance in Projection values VIP [54] and the cumulative variance explained by the top two PLSR components. The idea behind VIP measure is to accumulate the importance of each variable being reflected by the loading weights from each component. It is generally accepted that a variable should be selected if VIP > 1, but a proper threshold between 0.83 and 1.21 can yield more relevant variables [54,55]. A meteorological variable was declared important when 1) its variable importance in projection (VIP) was greater than 0.83 and 2) the cumulative variance explained by that meteorological observation by the top two PLSR components was at least 40%. The criterion to asses that the elimination of uninformative variables improves the model is to compare PRESS values obtained for the complete and the reduced model (new). If PRESS_new_ < PRESS we can conclude that the elimination of uninformative variables improve modeling.

All computation were performed using the PLS procedure of the SAS 9.3 Statistical Package (Base SAS® 9.3).

#### Spatial analysis method (SAM)

Other approach to assess the effect of any selection on polymorphisms across populations is the Spatial Analysis Method (SAM) developed by [56,57]. SAM is based on the *spatial coincidence* analysis to connect genetic information with geo-environmental data. The logistic regression uses random binomial variables as response for the model, thus, each allele is set to ‘1’ if it occurs in a given individual, and to ‘0’ if not. Logistic regression is used to assess the significance of the models constituted by all possible marker-environmental variable pairs. The comparison of observed with predicted values is based on the likelihood ratio (*G*) and Wald (*W*) tests [58] to determine the significance of the models. For both tests, the null hypothesis is that the model with the examined variable does not explain the observed distribution better than a model with a constant only. A model is considered significant only if both tests reject the corresponding null hypothesis. To restrict the analysis to robust candidate associations the Bonferroni correction was applied and only cumulated tests in which both *W* and *G* tests were significant were used to identify associated loci [56]. Computations were performed using the MatSAM v2Beta software [57].

### Statistical analysis to detect loci under selection across populations

#### Bayesian Test of Beaumont and Balding

The Bayesian test of Beaumont and Balding [59] evaluate the relationship between *F*_*ST*_ and *H*_*e*_ (expected heterozygosity) describing the expected distribution of Wright’s inbreeding coefficient *F*_*ST*_ vs. *H*_*e*_ under an island model of migration with neutral markers. This distribution is used to identify outlier loci that have excessively high or low *F*_*ST*_ compared to neutral expectations. Such outlier loci are candidates for being subject to selection [60]. Low *F*_*ST*_ outliers indicate loci subject to balancing selection, whereas high outliers suggest adaptative (directional) selection [59]. The Bayesian Test method of Beaumont and Balding was assessed using the LOSITAN (Looking for Selection In a Tangled dataset) package [60]. Initially 100,000 simulations under the infinite allele mutation model were run using all populations and all unlinked loci to determine a first candidate subset of selected loci in order to remove them from the computation of the neutral *F*_*ST*_. After the first run, all loci outside the desired confidence interval (99%) are removed. Subsequently a new 100,000 simulations run was developed to compute the mean neutral *F*_*ST*_. A final run of LOSITAN using all loci is then conducted using the last computed mean. Also, a frequentist method based on moment-based estimates of *F*_*ST*_, using the FDIST option of the LOSITAN package, was tested to compare results with the Bayesian approach.

### Phylogenetic Relationship between species from the Caprinae subfamily

Haplotype sequences from the different species analyzed were inferred by using PLINK software [45]. Promoter sequences were aligned by CLUSTAL. MEGA 6 software [61] was used to estimate nucleotide substitution models, evolutionary divergence and Tajima’s Neutrality Test and to construct the ML tree. Initial tree(s) for the heuristic search were obtained by applying the BioNJ method to a matrix of pairwise distances estimated using the Maximum Composite Likelihood (MCL) approach. A discrete Gamma distribution was used to model evolutionary rate differences among sites (5 categories (+*G*, parameter = 5.6803)).

## Availability of supporting data

The data sets supporting the results of this article are included as an additional file (AF9).

## Additional files

Additional file 1:
**Hardy Weinberg equilibrium test for all sheep breeds and for each breed separately.**
Additional file 2:
**Linkage disequilibrium of the 11 polymorphisms in all sheep breeds studied.**
Additional file 3:
**Population pairwise FSTs.**
Additional file 4:
**Basic statistics for polymorphisms MAF and environmental variables considered.**
Additional file 5:
**Pearson (above diagonal) and Spearman (below diagonal) correlations and significance for MAF of polymorphisms and environmental variables.** In bold and shading significant values at α = 0.05. Additional file 6:
**Regression coefficients of scaled and centered variables for predictors and responses.**
Additional file 7:
**Alignment of the 12 different species studied and their identity with the reference sequence (** ***Ovis aries*** **) based on their most frequent haplotype.** Also accession numbers of each sequence are shown. Additional file 8:
**Haplotypes inferred by PLINK for sheep breeds and wild species studied.**
Additional file 9:
**Genotypes of all animals used in this study for all the polymorphic sites detected in each species.**
