# Supplementary material for: Looking for adaptive footprints in the HSP90AA1 ovine gene
Source: BMC Evol Biol. 2015 Feb 4;15:7. doi: 10.1186/s12862-015-0280-x (PMC4351680; doi:10.1186/s12862-015-0280-x)
Supplement: Additional file 1: — Hardy Weinberg equilibrium test for all sheep breeds and for each breed separately. [file 12862_2015_280_MOESM1_ESM.docx]

**Additional File1 (AF1)** Hardy Weinberg equilibrium test for all sheep breeds and for each breed separately.

**All populations**

**CHR SNP TEST A1 A2 GENO O(HET) E(HET) P**

18 g.444A>G ALL A G 19/168/649 0.201 0.2161 0.05292

18 g.468G>T ALL G T 17/198/621 0.2368 0.239 0.7724

18 g.516_517insG ALL I D 13/143/680 0.1711 0.1817 0.08821

18 g-522A>G ALL A G 0/31/805 0.03708 0.03639 1

18 g-524G>T ALL G T 14/193/629 0.2309 0.2294 1

18 g.528G>A ALL G A 80/339/417 0.4055 0.4188 0.3639

18 g.601A>C ALL A C 17/197/622 0.2356 0.2381 0.7711

18 g.660G>C ALL G C 102/370/364 0.4426 0.4509 0.5918

**18 g.666_667insC ALL I D 32/94/710 0.1124 0.1711** **1.237e-015**

18 g.667_668insC ALL I D 43/291/502 0.3481 0.3493 0.9212

**18 g.703_704del(2)A ALL D I 145/345/346 0.4127 0.4711** **0.0004136**

**AKA**

**CHR SNP TEST A1 A2 GENO O(HET) E(HET) P**

18 g.444A>G ALL A G 0/6/17 0.2609 0.2268 1

18 g.468G>T ALL G T 0/9/14 0.3913 0.3147 0.5443

18 g.516_517insG ALL I D 0/1/22 0.04348 0.04253 1

18 g-522A>G ALL A G 0/2/21 0.08696 0.08318 1

18 g-524G>T ALL G T 0/9/14 0.3913 0.3147 0.5443

18 g.528G>A ALL G A 1/13/9 0.5652 0.4395 0.3453

18 g.601A>C ALL A C 1/9/13 0.3913 0.3639 1

18 g.660G>C ALL G C 3/12/8 0.5217 0.4764 1

18 g.666_667insC ALL I D 1/5/17 0.2174 0.258 0.4137

18 g.667_668insC ALL I D 0/11/12 0.4783 0.3639 0.2787

18 g.703_704del(2)A ALL D I 3/12/8 0.5217 0.4764 1

**ARME**

**CHR SNP TEST A1 A2 GENO O(HET) E(HET) P**

18 g.444A>G ALL A G 0/5/13 0.2778 0.2392 1

18 g.468G>T ALL G T 0/3/15 0.1667 0.1528 1

18 g.516_517insG ALL I D 0/7/11 0.3889 0.3133 1

18 g-522A>G ALL A G 0/0/18 0 0 1

18 g-524G>T ALL G T 0/3/15 0.1667 0.1528 1

18 g.528G>A ALL G A 1/8/9 0.4444 0.4012 1

18 g.601A>C ALL A C 0/3/15 0.1667 0.1528 1

18 g.660G>C ALL G C 1/9/8 0.5 0.4244 1

**18 g.666_667insC ALL I D 3/0/15 0 0.2778 0.0004189**

18 g.667_668insC ALL I D 0/9/9 0.5 0.375 0.524

18 g.703_704del(2)A ALL D I 2/9/7 0.5 0.4614 1

**AS**

**CHR SNP TEST A1 A2 GENO O(HET) E(HET) P**

18 g.444A>G ALL A G 1/5/24 0.1667 0.2061 0.3253

18 g.468G>T ALL G T 0/17/13 0.5667 0.4061 0.06534

18 g.516_517insG ALL I D 0/0/30 0 0 1

18 g-522A>G ALL A G 0/0/30 0 0 1

18 g-524G>T ALL G T 0/17/13 0.5667 0.4061 0.06534

18 g.528G>A ALL G A 0/6/24 0.2 0.18 1

18 g.601A>C ALL A C 0/17/13 0.5667 0.4061 0.06534

18 g.660G>C ALL G C 0/6/24 0.2 0.18 1

**18 g.666_667insC ALL I D 3/3/24 0.1 0.255 0.006523**

18 g.667_668insC ALL I D 4/16/10 0.5333 0.48 0.7113

18 g.703_704del(2)A ALL D I 0/6/24 0.2 0.18 1

**AW**

**CHR SNP TEST A1 A2 GENO O(HET) E(HET) P**

18 g.444A>G ALL A G 0/1/29 0.03333 0.03278 1

18 g.468G>T ALL G T 4/17/9 0.5667 0.4861 0.4721

18 g.516_517insG ALL I D 0/1/29 0.03333 0.03278 1

18 g-522A>G ALL A G 0/0/30 0 0 1

18 g-524G>T ALL G T 4/17/9 0.5667 0.4861 0.4721

18 g.528G>A ALL G A 0/8/22 0.2667 0.2311 1

18 g.601A>C ALL A C 4/17/9 0.5667 0.4861 0.4721

18 g.660G>C ALL G C 0/8/22 0.2667 0.2311 1

18 g.666_667insC ALL I D 0/0/30 0 0 1

18 g.667_668insC ALL I D 1/19/10 0.6333 0.455 0.05185

18 g.703_704del(2)A ALL D I 0/9/21 0.3 0.255 1

**BAJ**

**CHR SNP TEST A1 A2 GENO O(HET) E(HET) P**

18 g.444A>G ALL A G 0/4/18 0.1818 0.1653 1

18 g.468G>T ALL G T 0/7/15 0.3182 0.2676 1

18 g.516_517insG ALL I D 0/1/21 0.04545 0.04442 1

18 g-522A>G ALL A G 0/3/19 0.1364 0.1271 1

18 g-524G>T ALL G T 0/8/14 0.3636 0.2975 1

18 g.528G>A ALL G A 1/13/8 0.5909 0.4494 0.3376

18 g.601A>C ALL A C 0/8/14 0.3636 0.2975 1

18 g.660G>C ALL G C 3/14/5 0.6364 0.4959 0.3864

18 g.666_667insC ALL I D 0/0/22 0 0 1

18 g.667_668insC ALL I D 0/3/19 0.1364 0.1271 1

18 g.703_704del(2)A ALL I D 5/11/6 0.5 0.499 1

**BNI**

**CHR SNP TEST A1 A2 GENO O(HET) E(HET) P**

18 g.444A>G ALL A G 1/7/19 0.2593 0.2778 0.5488

18 g.468G>T ALL G T 1/5/21 0.1852 0.2257 0.3582

18 g.516_517insG ALL I D 0/4/23 0.1481 0.1372 1

18 g-522A>G ALL A G 0/3/24 0.1111 0.1049 1

18 g-524G>T ALL G T 0/4/23 0.1481 0.1372 1

18 g.528G>A ALL G A 3/13/11 0.4815 0.4561 1

18 g.601A>C ALL A C 0/6/21 0.2222 0.1975 1

18 g.660G>C ALL G C 4/15/8 0.5556 0.489 0.6971

18 g.666_667insC ALL I D 1/6/20 0.2222 0.2524 0.4538

18 g.667_668insC ALL I D 0/10/17 0.3704 0.3018 0.5488

18 g.703_704del(2)A ALL D I 4/15/8 0.5556 0.489 0.6971

**BOUJ**

**CHR SNP TEST A1 A2 GENO O(HET) E(HET) P**

18 g.444A>G ALL A G 0/4/20 0.1667 0.1528 1

18 g.468G>T ALL G T 0/6/18 0.25 0.2188 1

18 g.516_517insG ALL I D 0/5/19 0.2083 0.1866 1

18 g-522A>G ALL A G 0/3/21 0.125 0.1172 1

18 g-524G>T ALL G T 0/6/18 0.25 0.2188 1

18 g.528G>A ALL G A 2/8/14 0.3333 0.375 0.5964

18 g.601A>C ALL A C 0/6/18 0.25 0.2188 1

18 g.660G>C ALL G C 4/6/14 0.25 0.4132 0.06029

18 g.666_667insC ALL I D 0/3/21 0.125 0.1172 1

18 g.667_668insC ALL I D 0/11/13 0.4583 0.3533 0.2889

18 g.703_704del(2)A ALL D I 4/6/14 0.25 0.4132 0.06029

**BOZ**

**CHR SNP TEST A1 A2 GENO O(HET) E(HET) P**

18 g.444A>G ALL A G 1/4/19 0.1667 0.2188 0.298

18 g.468G>T ALL G T 0/5/19 0.2083 0.1866 1

18 g.516_517insG ALL I D 0/2/22 0.08333 0.07986 1

18 g-522A>G ALL A G 0/0/24 0 0 1

18 g-524G>T ALL G T 0/5/19 0.2083 0.1866 1

18 g.528G>A ALL G A 4/11/9 0.4583 0.4783 1

18 g.601A>C ALL A C 0/5/19 0.2083 0.1866 1

**18 g.660G>C ALL G C 8/7/9 0.2917 0.4991 0.04665**

**18 g.666_667insC ALL I D 2/3/19 0.125 0.2491 0.04734**

18 g.667_668insC ALL I D 1/9/14 0.375 0.3533 1

**18 g.703_704del(2)A ALL D I 8/7/9 0.2917 0.4991 0.04665**

**CAUC**

**CHR SNP TEST A1 A2 GENO O(HET) E(HET) P**

18 g.444A>G ALL A G 1/4/20 0.16 0.2112 0.2867

18 g.468G>T ALL G T 0/5/20 0.2 0.18 1

18 g.516_517insG ALL I D 3/8/14 0.32 0.4032 0.33

18 g-522A>G ALL A G 0/0/25 0 0 1

18 g-524G>T ALL G T 0/3/22 0.12 0.1128 1

18 g.528G>A ALL G A 2/11/12 0.44 0.42 1

18 g.601A>C ALL A C 0/5/20 0.2 0.18 1

18 g.660G>C ALL G C 3/10/12 0.4 0.4352 0.6626

**18 g.666_667insC ALL I D 3/0/22 0 0.2112 0.0001447**

18 g.667_668insC ALL I D 1/8/16 0.32 0.32 1

18 g.703_704del(2)A ALL D I 5/8/12 0.32 0.4608 0.187

**Ch**

**CHR SNP TEST A1 A2 GENO O(HET) E(HET) P**

18 g.444A>G ALL A G 1/8/14 0.3478 0.3403 1

18 g.468G>T ALL G T 0/6/17 0.2609 0.2268 1

18 g.516_517insG ALL I D 0/7/16 0.3043 0.258 1

18 g-522A>G ALL A G 0/5/18 0.2174 0.1938 1

18 g-524G>T ALL G T 0/6/17 0.2609 0.2268 1

18 g.528G>A ALL G A 0/12/11 0.5217 0.3856 0.2699

18 g.601A>C ALL A C 0/6/17 0.2609 0.2268 1

18 g.660G>C ALL G C 0/12/11 0.5217 0.3856 0.2699

18 g.666_667insC ALL I D 1/6/16 0.2609 0.2873 0.519

18 g.667_668insC ALL I D 2/7/14 0.3043 0.3639 0.5609

18 g.703_704del(2)A ALL D I 0/12/11 0.5217 0.3856 0.2699

**Cl**

**CHR SNP TEST A1 A2 GENO O(HET) E(HET) P**

18 g.444A>G ALL A G 0/1/25 0.03846 0.03772 1

18 g.468G>T ALL G T 0/4/22 0.1538 0.142 1

18 g.516_517insG ALL I D 0/1/25 0.03846 0.03772 1

18 g-522A>G ALL A G 0/0/26 0 0 1

18 g-524G>T ALL G T 0/4/22 0.1538 0.142 1

18 g.528G>A ALL G A 0/10/16 0.3846 0.3107 0.5449

18 g.601A>C ALL A C 0/3/23 0.1154 0.1087 1

18 g.660G>C ALL G C 0/10/16 0.3846 0.3107 0.5449

18 g.666_667insC ALL I D 0/0/26 0 0 1

18 g.667_668insC ALL D I 5/11/10 0.4231 0.4815 0.6827

18 g.703_704del(2)A ALL D I 0/11/15 0.4231 0.3336 0.5498

**Ct**

**CHR SNP TEST A1 A2 GENO O(HET) E(HET) P**

18 g.444A>G ALL A G 0/1/32 0.0303 0.02984 1

18 g.468G>T ALL G T 0/1/32 0.0303 0.02984 1

18 g.516_517insG ALL I D 3/15/15 0.4545 0.4339 1

18 g-522A>G ALL A G 0/0/33 0 0 1

18 g-524G>T ALL G T 0/1/32 0.0303 0.02984 1

18 g.528G>A ALL G A 5/14/14 0.4242 0.4628 0.7085

**18 g.601A>C ALL A C 1/0/32 0 0.05877 0.01538**

18 g.660G>C ALL G C 5/14/14 0.4242 0.4628 0.7085

18 g.666_667insC ALL I D 0/0/33 0 0 1

18 g.667_668insC ALL I D 0/6/27 0.1818 0.1653 1

18 g.703_704del(2)A ALL D I 8/12/13 0.3636 0.4885 0.1597

**DGL**

**CHR SNP TEST A1 A2 GENO O(HET) E(HET) P**

18 g.444A>G ALL A G 0/0/24 0 0 1

**18 g.468G>T ALL G T 3/3/18 0.125 0.3047 0.01309**

18 g.516_517insG ALL I D 0/1/23 0.04167 0.0408 1

18 g-522A>G ALL A G 0/0/24 0 0 1

**18 g-524G>T ALL G T 3/3/18 0.125 0.3047 0.01309**

18 g.528G>A ALL A G 4/10/10 0.4167 0.4688 0.6666

**18 g.601A>C ALL A C 3/3/18 0.125 0.3047 0.01309**

18 g.660G>C ALL C G 4/10/10 0.4167 0.4688 0.6666

18 g.666_667insC ALL I D 0/0/24 0 0 1

18 g.667_668insC ALL I D 0/2/22 0.08333 0.07986 1

18 g.703_704del(2)A ALL I D 4/10/10 0.4167 0.4688 0.6666

**EDIL**

**CHR SNP EST A1 A2 GENO O(HET) E(HET) P**

18 g.444A>G ALL A G 0/0/30 0 0 1

18 g.468G>T ALL G T 0/1/29 0.03333 0.03278 1

18 g.516_517insG ALL I D 0/3/27 0.1 0.095 1

18 g-522A>G ALL A G 0/1/29 0.03333 0.03278 1

18 g-524G>T ALL G T 0/1/29 0.03333 0.03278 1

**18 g.528G>A ALL A G 1/21/8 0.7 0.4728 0.01815**

18 g.601A>C ALL A C 0/1/29 0.03333 0.03278 1

**18 g.660G>C ALL C G 0/20/10 0.6667 0.4444 0.01082**

18 g.666_667insC ALL I D 0/0/30 0 0 1

18 g.667_668insC ALL I D 0/1/29 0.03333 0.03278 1

18 g.703_704del(2)A ALL I D 0/3/27 0.1 0.095 1

**IV**

**CHR SNP TEST A1 A2 GENO O(HET) E(HET) P**

18 g.444A>G ALL A G 1/1/13 0.06667 0.18 0.1034

18 g.468G>T ALL G T 2/5/8 0.3333 0.42 0.5394

18 g.516_517insG ALL I D 0/0/15 0 0 1

18 g-522A>G ALL A G 0/1/14 0.06667 0.06444 1

18 g-524G>T ALL G T 2/5/8 0.3333 0.42 0.5394

18 g.528G>A ALL G A 3/3/9 0.2 0.42 0.05807

18 g.601A>C ALL A C 2/5/8 0.3333 0.42 0.5394

18 g.660G>C ALL G C 3/4/8 0.2667 0.4444 0.2324

18 g.666_667insC ALL I D 1/1/13 0.06667 0.18 0.1034

18 g.667_668insC ALL I D 1/2/12 0.1333 0.2311 0.2031

18 g.703_704del(2)A ALL D I 3/4/8 0.2667 0.4444 0.2324

**KAR**

**CHR SNP TEST A1 A2 GENO O(HET) E(HET) P**

18 g.444A>G ALL A G 0/0/15 0 0 1

18 g.468G>T ALL G T 1/8/6 0.5333 0.4444 1

18 g.516_517insG ALL I D 0/0/15 0 0 1

18 g-522A>G ALL A G 0/0/15 0 0 1

18 g-524G>T ALL G T 0/9/6 0.6 0.42 0.2372

18 g.528G>A ALL A G 0/11/4 0.7333 0.4644 0.08526

18 g.601A>C ALL A C 0/9/6 0.6 0.42 0.2372

**18 g.660G>C ALL C G 0/12/3 0.8 0.48 0.02785**

18 g.666_667insC ALL I D 0/0/15 0 0 1

18 g.667_668insC ALL I D 0/0/15 0 0 1

18 g.703_704del(2)A ALL I D 0/11/4 0.7333 0.4644 0.08526

**KARM**

**CHR SNP TEST A1 A2 GENO O(HET) E(HET) P**

18 g.444A>G ALL A G 0/1/14 0.06667 0.06444 1

18 g.468G>T ALL G T 0/6/9 0.4 0.32 1

18 g.516_517insG ALL I D 0/1/14 0.06667 0.06444 1

18 g-522A>G ALL A G 0/0/15 0 0 1

18 g-524G>T ALL G T 0/6/9 0.4 0.32 1

18 g.528G>A ALL A G 1/10/4 0.6667 0.48 0.2889

18 g.601A>C ALL A C 0/6/9 0.4 0.32 1

18 g.660G>C ALL C G 0/11/4 0.7333 0.4644 0.08526

18 g.666_667insC ALL I D 0/0/15 0 0 1

**18 g.667_668insC ALL I D 1/0/14 0 0.1244 0.03448**

18 g.703_704del(2)A ALL I D 0/4/11 0.2667 0.2311 1

**KRB**

**CHR SNP TEST A1 A2 GENO O(HET) E(HET) P**

18 g.444A>G ALL A G 0/6/18 0.25 0.2188 1

18 g.468G>T ALL G T 0/6/18 0.25 0.2188 1

18 g.516_517insG ALL I D 0/0/24 0 0 1

18 g-522A>G ALL A G 0/2/22 0.08333 0.07986 1

18 g-524G>T ALL G T 0/4/20 0.1667 0.1528 1

18 g.528G>A ALL G A 3/17/4 0.7083 0.4991 0.09794

18 g.601A>C ALL A C 0/4/20 0.1667 0.1528 1

18 g.660G>C ALL C G 2/16/6 0.6667 0.4861 0.1117

18 g.666_667insC ALL I D 0/2/22 0.08333 0.07986 1

18 g.667_668insC ALL I D 0/10/14 0.4167 0.3299 0.5394

18 g.703_704del(2)A ALL I D 2/16/6 0.6667 0.4861 0.1117

**KRC**

**CHR SNP TEST A1 A2 GENO O(HET) E(HET) P**

18 g.444A>G ALL A G 0/5/23 0.1786 0.1626 1

18 g.468G>T ALL G T 0/8/20 0.2857 0.2449 1

18 g.516_517insG ALL I D 0/1/27 0.03571 0.03508 1

18 g-522A>G ALL A G 0/1/27 0.03571 0.03508 1

18 g-524G>T ALL G T 0/8/20 0.2857 0.2449 1

18 g.528G>A ALL A G 4/17/7 0.6071 0.4943 0.4413

18 g.601A>C ALL A C 0/8/20 0.2857 0.2449 1

18 g.660G>C ALL C G 4/17/7 0.6071 0.4943 0.4413

18 g.666_667insC ALL I D 1/2/25 0.07143 0.1327 0.1081

18 g.667_668insC ALL I D 0/7/21 0.25 0.2188 1

18 g.703_704del(2)A ALL I D 3/15/10 0.5357 0.4688 0.6891

**KRY**

**CHR SNP TEST A1 A2 GENO O(HET) E(HET) P**

18 g.444A>G ALL A G 0/3/19 0.1364 0.1271 1

18 g.468G>T ALL G T 2/8/12 0.3636 0.3967 0.6232

18 g.516_517insG ALL I D 0/6/16 0.2727 0.2355 1

18 g-522A>G ALL A G 0/1/21 0.04545 0.04442 1

18 g-524G>T ALL G T 1/8/13 0.3636 0.3512 1

18 g.528G>A ALL G A 2/6/14 0.2727 0.3512 0.2712

18 g.601A>C ALL A C 2/7/13 0.3182 0.375 0.5683

18 g.660G>C ALL G C 2/8/12 0.3636 0.3967 0.6232

18 g.666_667insC ALL I D 1/1/20 0.04545 0.1271 0.06977

18 g.667_668insC ALL I D 0/4/18 0.1818 0.1653 1

18 g.703_704del(2)A ALL D I 2/8/12 0.3636 0.3967 0.6232

**KVR**

**CHR SNP TEST A1 A2 GENO O(HET) E(HET) P**

18 g.444A>G ALL A G 0/3/13 0.1875 0.1699 1

18 g.468G>T ALL G T 0/3/13 0.1875 0.1699 1

18 g.516_517insG ALL I D 0/1/15 0.0625 0.06055 1

18 g-522A>G ALL A G 0/0/16 0 0 1

18 g-524G>T ALL G T 0/2/14 0.125 0.1172 1

18 g.528G>A ALL G A 1/5/10 0.3125 0.3418 1

18 g.601A>C ALL A C 0/3/13 0.1875 0.1699 1

18 g.660G>C ALL G C 0/8/8 0.5 0.375 0.5127

18 g.666_667insC ALL I D 0/2/14 0.125 0.1172 1

18 g.667_668insC ALL I D 2/10/4 0.625 0.4922 0.6086

18 g.703_704del(2)A ALL D I 0/8/8 0.5 0.375 0.5127

**L**

**CHR SNP TEST A1 A2 GENO O(HET) E(HET) P**

18 g.444A>G ALL A G 3/11/16 0.3667 0.4061 0.6554

18 g.468G>T ALL G T 1/1/28 0.03333 0.095 0.05085

18 g.516_517insG ALL I D 0/1/29 0.03333 0.03278 1

18 g-522A>G ALL A G 0/0/30 0 0 1

18 g-524G>T ALL G T 1/1/28 0.03333 0.095 0.05085

18 g.528G>A ALL G A 3/14/13 0.4667 0.4444 1

18 g.601A>C ALL A C 1/1/28 0.03333 0.095 0.05085

18 g.660G>C ALL G C 5/15/10 0.5 0.4861 1

18 g.666_667insC ALL I D 3/11/16 0.3667 0.4061 0.6554

18 g.667_668insC ALL I D 3/11/16 0.3667 0.4061 0.6554

18 g.703_704del(2)A ALL D I 5/15/10 0.5 0.4861 1

**LX**

**CHR SNP TEST A1 A2 GENO O(HET) E(HET) P**

18 g.444A>G ALL A G 0/19/22 0.4634 0.356 0.08547

18 g.468G>T ALL G T 0/12/29 0.2927 0.2499 0.5704

18 g.516_517insG ALL I D 1/15/25 0.3659 0.3287 0.6633

18 g-522A>G ALL A G 0/0/41 0 0 1

18 g-524G>T ALL G T 0/12/29 0.2927 0.2499 0.5704

18 g.528G>A ALL G A 0/8/33 0.1951 0.1761 1

18 g.601A>C ALL A C 0/12/29 0.2927 0.2499 0.5704

18 g.660G>C ALL G C 0/9/32 0.2195 0.1954 1

18 g.666_667insC ALL I D 0/15/26 0.3659 0.2989 0.3121

18 g.667_668insC ALL I D 0/20/21 0.4878 0.3688 0.08155

18 g.703_704del(2)A ALL D I 1/19/21 0.4634 0.381 0.2448

**MAN**

**CHR SNP TEST A1 A2 GENO O(HET) E(HET) P**

18 g.444A>G ALL A G 3/10/13 0.3846 0.426 0.6526

18 g.468G>T ALL G T 2/10/14 0.3846 0.3935 1

18 g.516_517insG ALL I D 2/7/17 0.2692 0.3336 0.2878

18 g-522A>G ALL A G 0/0/26 0 0 1

18 g-524G>T ALL G T 2/8/16 0.3077 0.355 0.5783

18 g.528G>A ALL G A 0/1/25 0.03846 0.03772 1

18 g.601A>C ALL A C 2/10/14 0.3846 0.3935 1

18 g.660G>C ALL G C 1/13/12 0.5 0.4105 0.6293

18 g.666_667insC ALL I D 0/2/24 0.07692 0.07396 1

18 g.667_668insC ALL I D 0/2/24 0.07692 0.07396 1

18 g.703_704del(2)A ALL D I 2/12/12 0.4615 0.426 1

**ME**

**CHR SNP TEST A1 A2 GENO O(HET) E(HET) P**

18 g.444A>G ALL A G 1/12/16 0.4138 0.3662 1

18 g.468G>T ALL G T 0/4/25 0.1379 0.1284 1

18 g.516_517insG ALL I D 0/5/24 0.1724 0.1576 1

18 g-522A>G ALL A G 0/3/26 0.1034 0.0981 1

18 g-524G>T ALL G T 0/4/25 0.1379 0.1284 1

18 g.528G>A ALL G A 1/6/22 0.2069 0.2378 0.4268

18 g.601A>C ALL A C 0/4/25 0.1379 0.1284 1

18 g.660G>C ALL G C 2/12/15 0.4138 0.3995 1

18 g.666_667insC ALL I D 1/3/25 0.1034 0.1576 0.1707

18 g.667_668insC ALL I D 2/14/13 0.4828 0.4281 0.6788

18 g.703_704del(2)A ALL D I 5/9/15 0.3103 0.4405 0.1122

**MNCH**

**CHR SNP TEST A1 A2 GENO O(HET) E(HET) P**

18 g.444A>G ALL A G 0/6/54 0.1 0.095 1

18 g.468G>T ALL G T 0/19/41 0.3167 0.2665 0.3301

18 g.516_517insG ALL I D 1/20/39 0.3333 0.2994 0.6693

18 g-522A>G ALL A G 0/2/58 0.03333 0.03278 1

18 g-524G>T ALL G T 0/19/41 0.3167 0.2665 0.3301

18 g.528G>A ALL G A 2/32/26 0.5333 0.42 0.06243

18 g.601A>C ALL A C 0/20/40 0.3333 0.2778 0.3395

18 g.660G>C ALL G C 3/32/25 0.5333 0.4328 0.1323

18 g.666_667insC ALL I D 0/4/56 0.06667 0.06444 1

18 g.667_668insC ALL I D 1/25/34 0.4167 0.3488 0.2614

18 g.703_704del(2)A ALL D I 3/32/25 0.5333 0.4328 0.1323

**OL**

**CHR SNP TEST A1 A2 GENO O(HET) E(HET) P**

18 g.444A>G ALL A G 2/13/15 0.4333 0.4061 1

18 g.468G>T ALL G T 0/0/30 0 0 1

18 g.516_517insG ALL I D 0/5/25 0.1667 0.1528 1

18 g-522A>G ALL A G 0/0/30 0 0 1

18 g-524G>T ALL G T 0/0/30 0 0 1

18 g.528G>A ALL G A 3/14/13 0.4667 0.4444 1

18 g.601A>C ALL A C 0/0/30 0 0 1

18 g.660G>C ALL G C 3/14/13 0.4667 0.4444 1

18 g.666_667insC ALL I D 2/11/17 0.3667 0.375 1

18 g.667_668insC ALL I D 1/15/14 0.5 0.4061 0.3755

18 g.703_704del(2)A ALL D I 3/14/13 0.4667 0.4444 1

**PRAM**

**CHR SNP TEST A1 A2 GENO O(HET) E(HET) P**

18 g.444A>G ALL A G 1/14/14 0.4828 0.3995 0.3899

18 g.468G>T ALL G T 0/9/20 0.3103 0.2622 1

18 g.516_517insG ALL I D 0/3/26 0.1034 0.0981 1

18 g-522A>G ALL A G 0/0/29 0 0 1

18 g-524G>T ALL G T 0/9/20 0.3103 0.2622 1

**18 g.528G>A ALL G A 6/6/17 0.2069 0.4281 0.007234**

18 g.601A>C ALL A C 0/9/20 0.3103 0.2622 1

**18 g.660G>C ALL G C 6/7/16 0.2414 0.4405 0.02826**

18 g.666_667insC ALL I D 3/10/16 0.3448 0.3995 0.6385

18 g.667_668insC ALL I D 4/12/13 0.4138 0.4518 0.687

**18 g.703_704del(2)A ALL D I 6/7/16 0.2414 0.4405 0.02826**

**RA**

**CHR SNP TEST A1 A2 GENO O(HET) E(HET) P**

18 g.444A>G ALL A G 2/8/32 0.1905 0.2449 0.1724

18 g.468G>T ALL G T 1/7/34 0.1667 0.1913 0.3799

18 g.516_517insG ALL I D 2/13/27 0.3095 0.3228 0.6583

18 g-522A>G ALL A G 0/4/38 0.09524 0.0907 1

18 g-524G>T ALL G T 1/7/34 0.1667 0.1913 0.3799

18 g.528G>A ALL G A 2/10/30 0.2381 0.2778 0.299

18 g.601A>C ALL A C 1/7/34 0.1667 0.1913 0.3799

18 g.660G>C ALL G C 2/13/27 0.3095 0.3228 0.6583

**18 g.666_667insC ALL I D 3/3/36 0.07143 0.1913 0.002303**

18 g.667_668insC ALL I D 3/17/22 0.4048 0.3977 1

18 g.703_704del(2)A ALL D I 2/14/26 0.3333 0.3367 1

**SZ**

**CHR SNP T EST A1 A2 GENO O(HET) E(HET) P**

18 g.444A>G ALL A G 0/0/26 0 0 1

18 g.468G>T ALL G T 0/2/24 0.07692 0.07396 1

18 g.516_517insG ALL I D 0/0/26 0 0 1

18 g-522A>G ALL A G 0/0/26 0 0 1

18 g-524G>T ALL G T 0/3/23 0.1154 0.1087 1

18 g.528G>A ALL G A 0/8/18 0.3077 0.2604 1

18 g.601A>C ALL A C 0/3/23 0.1154 0.1087 1

18 g.660G>C ALL G C 1/10/15 0.3846 0.355 1

18 g.666_667insC ALL I D 0/0/26 0 0 1

18 g.667_668insC ALL I D 5/10/11 0.3846 0.4734 0.4094

18 g.703_704del(2)A ALL D I 1/10/15 0.3846 0.355 1

**VdB**

**CHR SNP TEST A1 A2 GENO O(HET) E(HET) P**

18 g.444A>G ALL A G 1/6/22 0.2069 0.2378 0.4268

18 g.468G>T ALL G T 0/1/28 0.03448 0.03389 1

18 g.516_517insG ALL I D 1/9/19 0.3103 0.3074 1

18 g-522A>G ALL A G 0/0/29 0 0 1

18 g-524G>T ALL G T 0/1/28 0.03448 0.03389 1

18 g.528G>A ALL G A 2/13/14 0.4483 0.4144 1

18 g.601A>C ALL A C 0/0/29 0 0 1

18 g.660G>C ALL G C 3/16/10 0.5517 0.4709 0.4575

**18 g.666_667insC ALL I D 3/1/25 0.03448 0.2122 0.0006319**

18 g.667_668insC ALL I D 1/9/19 0.3103 0.3074 1

18 g.703_704del(2)A ALL D I 4/16/9 0.5517 0.4851 0.7026

SNP SNP identifier

TEST Code indicating sample

A1 Minor allele code

A2 Major allele code

GENO Genotype counts: 11/12/22

O(HET) Observed heterozygosity

E(HET) Expected heterozygosity

P H-W p-value
