# Supplementary material for: Looking for adaptive footprints in the HSP90AA1 ovine gene
Source: BMC Evol Biol. 2015 Feb 4;15:7. doi: 10.1186/s12862-015-0280-x (PMC4351680; doi:10.1186/s12862-015-0280-x)
Supplement: Additional file 4: — Basic statistics for polymorphisms MAF and environmental variables considered. [file 12862_2015_280_MOESM4_ESM.docx]

**Additional File4 (AF4)** Basic statistics for polymorphisms MAF and environmental variables considered.

| **Variable** | **N** | **Mean** | **SD** | **Minimum** | **Maximun** |
| --- | --- | --- | --- | --- | --- |
| I_-668_ | 31 | 0.219 | 0.136 | 0 | 0.596 |
| I_-667_ | 31 | 0.124 | 0.130 | 0 | 0.417 |
| G_-660_ | 31 | 0.368 | 0.157 | 0.1 | 0.667 |
| A_-601_ | 31 | 0.144 | 0.100 | 0 | 0.417 |
| A_-522_ | 31 | 0.019 | 0.028 | 0 | 0.109 |
| I_-516_ | 31 | 0.089 | 0.092 | 0 | 0.318 |
| LAT | 31 | 41.069 | 6.974 | 30.46 | 62.31 |
| LON | 31 | 26.783 | 27.788 | -6.96 | 107.43 |
| MINaT | 31 | 1.806 | 8.636 | -18.8 | 14 |
| MThm | 31 | 34.258 | 5.053 | 20.8 | 42 |
| ANT | 31 | 12.597 | 5.419 | 0 | 20.7 |
| TW | 31 | 21.906 | 6.784 | 13.4 | 36.5 |
| TAR | 31 | 484.903 | 175.813 | 110 | 782 |
| MxR | 31 | 77.645 | 37.075 | 19 | 153 |
| MiR | 31 | 13.774 | 12.395 | 0 | 42 |
| HrA | 31 | 66.548 | 7.593 | 41 | 80 |
