# Supplementary material for: Looking for adaptive footprints in the HSP90AA1 ovine gene
Source: BMC Evol Biol. 2015 Feb 4;15:7. doi: 10.1186/s12862-015-0280-x (PMC4351680; doi:10.1186/s12862-015-0280-x)
Supplement: Additional file 5: — Pearson (above diagonal) and Spearman (below diagonal) correlations and significance for MAF of polymorphisms and environmental variables. In bold and shading significant values at α = 0.05. [file 12862_2015_280_MOESM5_ESM.docx]

**Additional File5 (AF5)** Pearson (above diagonal) and Spearman (below diagonal) correlations and significance for MAF of polymorphisms and environmental variables. In bold and shading significant values at α=0.05.

|  | **I_-668_** | **I_-667_** | **G_-660_** | **A_-601_** | **A_-522_** | **I_-516_** | **LAT** | **LON** | **MINaT** | **MThm** | **ANT** | **TW** | **TAR** | **MxR** | **MiR** | **HrA** |
| --- | --- | --- | --- | --- | --- | --- | --- | --- | --- | --- | --- | --- | --- | --- | --- | --- |
| **I_-668_** | **1** | 0.2800 | **-0.6830** | -0.1923 | -0.1059 | -0.1844 | -0.2435 | -0.3188 | **0.4686** | -0.1196 | **0.4040** | **-0.5187** | **0.4519** | **0.5689** | -0.0595 | 0.1153 |
|  |  | 0.1272 | <.0001 | 0.3000 | 0.5709 | 0.3208 | 0.1869 | 0.0805 | 0.0078 | 0.5217 | 0.0242 | 0.0028 | 0.0107 | 0.0008 | 0.7507 | 0.5369 |
| **I-_667_** | **0.4306** | **1** | -0.2433 | -0.2016 | 0.1083 | 0.0164 | 0.2718 | -0.2800 | -0.0139 | **-0.5030** | -0.1433 | -0.1913 | **0.3756** | 0.0636 | **0.5513** | 0.3143 |
|  | 0.0156 |  | 0.1871 | 0.2769 | 0.5620 | 0.9304 | 0.1392 | 0.1271 | 0.9408 | 0.0039 | 0.4420 | 0.3026 | 0.0373 | 0.7340 | 0.0013 | 0.0851 |
| **G_-660_** | **-0.7069** | -0.1770 | **1** | -0.0999 | 0.0675 | -0.3086 | **0.3613** | **0.4172** | **-0.6003** | 0.0779 | **-0.5095** | **0.6543** | **-0.4251** | **-0.4978** | -0.0267 | 0.0454 |
|  | <.0001 | 0.3409 |  | 0.5928 | 0.7181 | 0.0912 | 0.0458 | 0.0195 | 0.0004 | 0.6769 | 0.0034 | <.0001 | 0.0171 | 0.0044 | 0.8867 | 0.8082 |
| **A_-601_** | -0.2535 | -0.1182 | -0.0155 | **1** | -0.0027 | -0.2905 | -0.3522 | 0.1207 | 0.1680 | 0.3225 | 0.2332 | -0.0496 | -0.2305 | 0.0487 | -0.2764 | -0.2156 |
|  | 0.1688 | 0.5264 | 0.9339 |  | 0.9886 | 0.1129 | 0.0520 | 0.5177 | 0.3664 | 0.0769 | 0.2068 | 0.7912 | 0.2122 | 0.7947 | 0.1322 | 0.2442 |
| **A_-522_** | -0.1734 | 0.1680 | 0.1596 | 0.1118 | **1** | -0.0113 | -0.1178 | -0.0884 | -0.0323 | 0.2500 | -0.0721 | 0.0069 | -0.1836 | -0.1947 | -0.1739 | -0.1562 |
|  | 0.3509 | 0.3664 | 0.3911 | 0.5494 |  | 0.9519 | 0.5281 | 0.6364 | 0.8632 | 0.1750 | 0.7001 | 0.9705 | 0.3229 | 0.2939 | 0.3495 | 0.4015 |
| **I_-516_** | -0.1583 | 0.1314 | -0.2276 | -0.2735 | 0.0406 | **1** | -0.0326 | **-0.3682** | 0.1603 | -0.0918 | 0.1087 | -0.1912 | -0.0621 | -0.3390 | 0.2320 | -0.1905 |
|  | 0.3951 | 0.4810 | 0.2181 | 0.1366 | 0.8283 |  | 0.8619 | 0.0415 | 0.3889 | 0.6234 | 0.5606 | 0.3029 | 0.7399 | 0.0621 | 0.2092 | 0.3046 |
| **LAT** | -0.2568 | -0.0035 | 0.3525 | -0.2515 | -0.1486 | 0.1115 | **1** | **0.4584** | **-0.7916** | **-0.4957** | **-0.8416** | **0.5985** | 0.1697 | -0.0628 | **0.6432** | **0.6509** |
|  | 0.1632 | 0.9852 | 0.0518 | 0.1722 | 0.4250 | 0.5504 |  | 0.0095 | <.0001 | 0.0046 | <.0001 | 0.0004 | 0.3613 | 0.7370 | <.0001 | <.0001 |
| **LON** | -0.2961 | -0.2838 | **0.4331** | 0.1543 | -0.0713 | **-0.4429** | **0.4309** | **1** | **-0.6990** | -0.1364 | **-0.5800** | **0.7575** | -0.0745 | 0.1576 | 0.0373 | 0.2687 |
|  | 0.1058 | 0.1218 | 0.0149 | 0.4072 | 0.7032 | 0.0126 | 0.0155 |  | <.0001 | 0.4643 | 0.0006 | <.0001 | 0.6902 | 0.3971 | 0.8423 | 0.1438 |
| **MINaT** | **0.4047** | 0.0541 | **-0.5989** | 0.0911 | -0.0187 | 0.0976 | **-0.8265** | **-0.6167** | **1** | 0.2193 | **0.9686** | **-0.9185** | 0.1369 | 0.2202 | -0.3546 | **-0.3628** |
|  | 0.0239 | 0.7725 | 0.0004 | 0.6260 | 0.9206 | 0.6013 | <.0001 | 0.0002 |  | 0.2359 | <.0001 | <.0001 | 0.4627 | 0.2339 | 0.0503 | 0.0449 |
| **MThm** | -0.1584 | **-0.3587** | 0.0342 | 0.2854 | 0.2032 | -0.2318 | **-0.4335** | -0.0942 | 0.3028 | **1** | 0.3226 | -0.0083 | **-0.4774** | -0.1796 | **-0.5551** | **-0.4996** |
|  | 0.3947 | 0.0475 | 0.8553 | 0.1197 | 0.2728 | 0.2096 | 0.0148 | 0.6143 | 0.0978 |  | 0.0767 | 0.9645 | 0.0066 | 0.3337 | 0.0012 | 0.0042 |
| **ANT** | 0.3379 | 0.0104 | **-0.4756** | 0.1534 | -0.0074 | -0.0134 | **-0.8883** | **-0.4764** | **0.9539** | **0.3909** | **1** | **-0.7970** | 0.0026 | 0.1677 | **-0.4797** | **-0.4815** |
|  | 0.0630 | 0.9557 | 0.0069 | 0.4099 | 0.9684 | 0.9431 | <.0001 | 0.0067 | <.0001 | 0.0297 |  | <.0001 | 0.9889 | 0.3671 | 0.0063 | 0.0061 |
| **TW** | **-0.4840** | -0.1867 | **0.6948** | -0.0299 | 0.0333 | -0.1838 | **0.6333** | **0.6493** | **-0.9053** | -0.0902 | **-0.7637** | **1** | -0.3429 | -0.3045 | 0.0831 | 0.0840 |
|  | 0.0058 | 0.3145 | <.0001 | 0.8733 | 0.8589 | 0.3224 | 0.0001 | <.0001 | <.0001 | 0.6296 | <.0001 |  | 0.0590 | 0.0958 | 0.6569 | 0.6533 |
| **TAR** | **0.5034** | 0.2857 | **-0.4531** | -0.2428 | -0.2844 | 0.0081 | 0.1322 | -0.1810 | 0.1602 | **-0.5478** | 0.0077 | **-0.3844** | **1** | **0.7753** | **0.5097** | **0.5126** |
|  | 0.0039 | 0.1193 | 0.0105 | 0.1882 | 0.1210 | 0.9655 | 0.4785 | 0.3299 | 0.3893 | 0.0014 | 0.9673 | 0.0327 |  | <.0001 | 0.0034 | 0.0032 |
| **MxR** | **0.5828** | 0.0812 | **-0.4637** | -0.1028 | -0.2442 | -0.3231 | -0.0505 | 0.0591 | 0.2323 | -0.2611 | 0.1451 | -0.3444 | **0.7966** | **1** | 0.0300 | **0.3589** |
|  | 0.0006 | 0.6640 | 0.0086 | 0.5823 | 0.1855 | 0.0762 | 0.7875 | 0.7520 | 0.2085 | 0.1559 | 0.4360 | 0.0578 | <.0001 |  | 0.8727 | 0.0474 |
| **MiR** | -0.0404 | 0.2537 | 0.0934 | -0.3177 | -0.1906 | 0.3225 | **0.8055** | 0.1160 | **-0.5621** | **-0.6118** | **-0.6832** | 0.2723 | **0.4183** | 0.0037 | **1** | **0.5489** |
|  | 0.8290 | 0.1685 | 0.6174 | 0.0816 | 0.3044 | 0.0768 | <.0001 | 0.5343 | 0.0010 | 0.0003 | <.0001 | 0.1384 | 0.0192 | 0.9841 |  | 0.0014 |
| **HrA** | -0.0151 | 0.0927 | 0.0319 | -0.0165 | -0.3084 | -0.0859 | **0.6155** | 0.2183 | -0.3364 | **-0.4711** | **-0.4840** | 0.0677 | **0.4245** | 0.3312 | **0.5365** | **1** |
|  | 0.9359 | 0.6199 | 0.8646 | 0.9299 | 0.0914 | 0.6459 | 0.0002 | 0.2381 | 0.0643 | 0.0075 | 0.0058 | 0.7173 | 0.0173 | 0.0688 | 0.0019 |  |
