# Supplementary material for: Looking for adaptive footprints in the HSP90AA1 ovine gene
Source: BMC Evol Biol. 2015 Feb 4;15:7. doi: 10.1186/s12862-015-0280-x (PMC4351680; doi:10.1186/s12862-015-0280-x)
Supplement: Additional file 6: — Regression coefficients of scaled and centered variables for predictors and responses. [file 12862_2015_280_MOESM6_ESM.docx]

**Additional File6 (AF6)** Regression coefficients of scaled and centered variables for predictors and responses.

|  | **g.667_668insC** | **g.666_667insC** | **g660G>C** | **g.601A>C** | **g.522A>G** | **g.516_517insG** |
| --- | --- | --- | --- | --- | --- | --- |
| **LAT** | -0.065 | 0.123 | 0.072 | -0.099 | -0.023 | 0.047 |
| **LON** | -0.017 | -0.251 | 0.103 | 0.129 | -0.008 | -0.290 |
| **MINaT** | 0.127 | 0.029 | -0.156 | 0.019 | -0.009 | 0.043 |
| **MThm** | -0.041 | -0.236 | 0.061 | 0.143 | 0.051 | -0.120 |
| **ANT** | 0.113 | -0.035 | -0.130 | 0.054 | -0.001 | -0.007 |
| **TW** | -0.141 | -0.115 | 0.180 | 0.032 | 0.025 | -0.093 |
| **TAR** | 0.177 | 0.116 | -0.163 | -0.047 | -0.078 | -0.042 |
| **MxR** | 0.264 | -0.113 | -0.190 | 0.099 | -0.091 | -0.293 |
| **MiR** | -0.020 | 0.279 | -0.023 | -0.174 | -0.030 | 0.207 |
| **HrA** | 0.080 | 0.043 | -0.041 | -0.029 | -0.061 | -0.099 |

LAT=latitude

LON=longitude

MINaT=minimum average temperature

MThm=maximum temperature of the hottest month

ANT=average annual temperature

TW (MAXaT- MINaT)=thermal width

TAR = total annual rainfall

MxR = maximum rainfall

MiR = minimum rainfall

HrA = relative average annual humidity (%)
