# Supplementary material for: Looking for adaptive footprints in the HSP90AA1 ovine gene
Source: BMC Evol Biol. 2015 Feb 4;15:7. doi: 10.1186/s12862-015-0280-x (PMC4351680; doi:10.1186/s12862-015-0280-x)
Supplement: Additional file 7: — Alignment of the 12 different species studied and their identity with the reference sequence ( Ovis aries ) based on their most frequent haplotype. Also accession numbers of each sequence are shown. [file 12862_2015_280_MOESM7_ESM.docx]

**Additional File7 (AF7)** Alignment of the 12 different species studied and their identity with the reference sequence (*Ovis aries*) based on their most frequent haplotype. Also accession numbers of each sequence are shown.


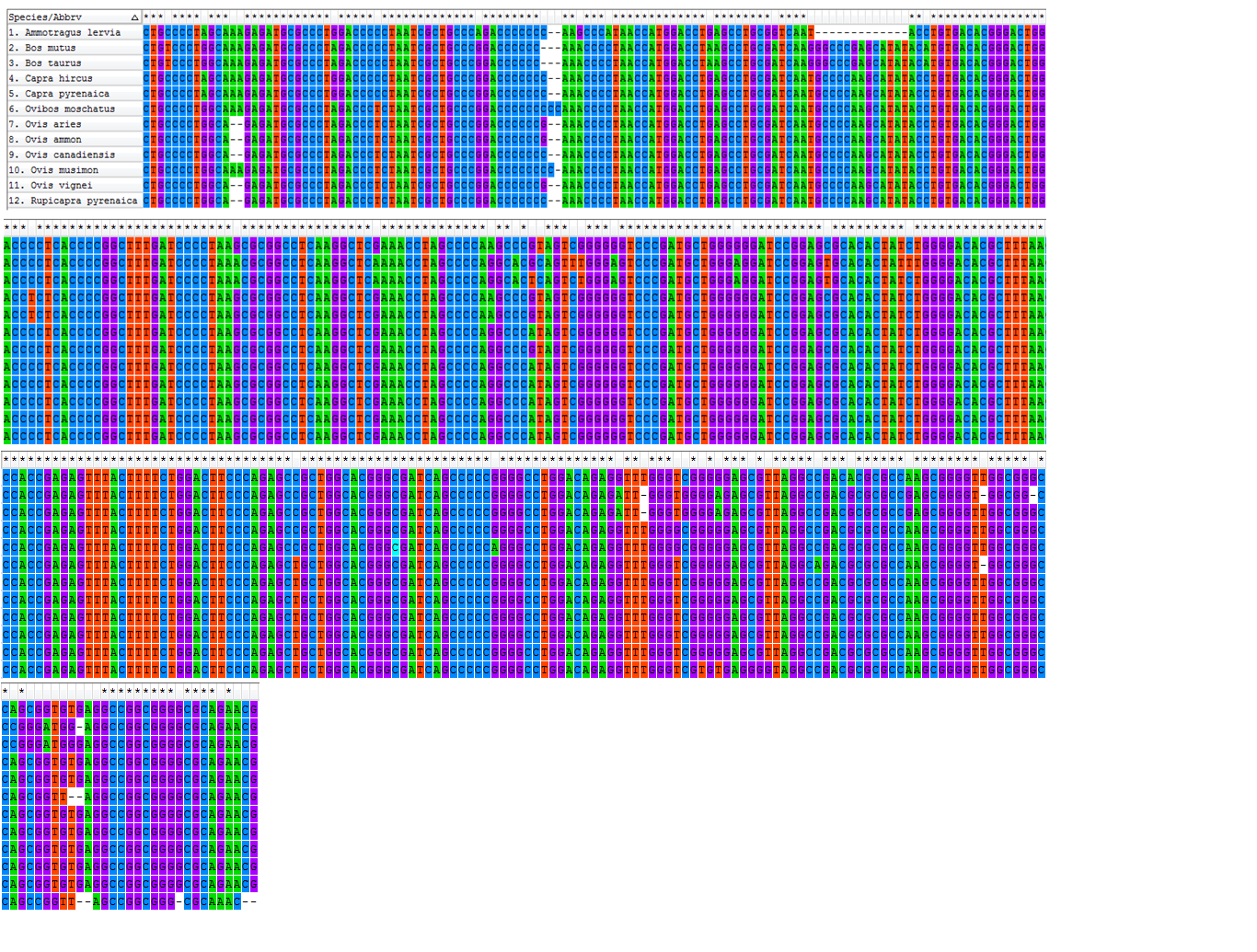


94% KP162093

92% KP162101

93% KP242296

98% KP162095

97% KP162096

98% KP162098

**100% DQ983231**

99% KP162092

99% KP162094

99% KP162097

99% KP162100

97% KP162099
