# Supplementary material for: Looking for adaptive footprints in the HSP90AA1 ovine gene
Source: BMC Evol Biol. 2015 Feb 4;15:7. doi: 10.1186/s12862-015-0280-x (PMC4351680; doi:10.1186/s12862-015-0280-x)
Supplement: Additional file 8: — Haplotypes inferred by PLINK for sheep breeds and wild species studied. [file 12862_2015_280_MOESM8_ESM.docx]

**Additional File8 (AF8)** Haplotypes inferred by PLINK for sheep breeds and wild species studied.

|  | | |  | | **Haplotype** | | | | | | | | | | | | | | | | | | | | | | | | | | | | | | | | | | | | | | | | | | | | | | | | | | | | | | | | | | | | | | | | | | | | | | | | | | | |
| --- | --- | --- | --- | --- | --- | --- | --- | --- | --- | --- | --- | --- | --- | --- | --- | --- | --- | --- | --- | --- | --- | --- | --- | --- | --- | --- | --- | --- | --- | --- | --- | --- | --- | --- | --- | --- | --- | --- | --- | --- | --- | --- | --- | --- | --- | --- | --- | --- | --- | --- | --- | --- | --- | --- | --- | --- | --- | --- | --- | --- | --- | --- | --- | --- | --- | --- | --- | --- | --- | --- | --- | --- | --- | --- | --- | --- | --- | --- | --- | --- |
| ***O. aries*** | | | **2N** | | H1 | | | H2 | | | H3 | H4 | | | H5 | | H6 | | H7 | | | H8 | | H9 | | | H10 | | | H11 | | H12 | | | | H13 | | | H14 | | H15 | | H16 | | | H17 | H18 | H19 | | | H20 | | H21 | | H22 | | | H23 | | H24 | | H25 | H26 | | H27 | | H28 | | H29 | | | H30 | H31 | H32 | H33 | | H34 | H35 | H36 | |
| AKA | | | 46 | | 15 | | | 6 | | | 4 | 9 | | | 1 | | 6 | | 3 | | | - | | 2 | | | - | | | - | | - | | | | - | | | - | | - | | - | | | - | - | - | | | - | | - | | - | | | - | | - | | - | - | | - | | - | | - | | | - | - | - | - | | - | - | - | |
| ARME | | | 36 | | 10 | | | 9 | | | 1 | 3 | | | 7 | | 3 | | - | | | 1 | | - | | | 1 | | | - | | 1 | | | | - | | | - | | - | | - | | | - | - | - | | | - | | - | | - | | | - | | - | | - | - | | - | | - | | - | | | - | - | - | - | | - | - | - | |
| AS | | | 60 | | 6 | | | 21 | | | 8 | 17 | | | - | | 6 | | - | | | 1 | | - | | | - | | | 1 | | - | | | | - | | | - | | - | | - | | | - | - | - | | | - | | - | | - | | | - | | - | | - | - | | - | | - | | - | | | - | - | - | - | | - | - | - | |
| AW | | | 60 | | 8 | | | 20 | | | 4 | 25 | | | 1 | | - | | - | | | 1 | | - | | | - | | | - | | - | | | | - | | | 1 | | - | | - | | | - | - | - | | | - | | - | | - | | | - | | - | | - | - | | - | | - | | - | | | - | - | - | - | | - | - | - | |
| BAJ | | | 44 | | 15 | | | 3 | | | 3 | 8 | | | - | | - | | 4 | | | 3 | | 3 | | | - | | | 3 | | 1 | | | | - | | | - | | - | | - | | | - | - | - | | | - | | - | | - | | | - | | - | | - | - | | - | | - | | - | | | - | - | - | - | | - | 1 | - | |
| BNI | | | 54 | | 19 | | | 4 | | | 6 | 4 | | | 4 | | 7 | | 2 | | | - | | 3 | | | 1 | | | 1 | | - | | | | 2 | | | - | | - | | - | | | - | 1 | - | | | - | | - | | - | | | - | | - | | - | - | | - | | - | | - | | | - | - | - | - | | - | - | - | |
| BOUJ | | | 48 | | 12 | | | 9 | | | 9 | 6 | | | 5 | | 2 | | - | | | - | | 3 | | | 2 | | | - | | - | | | | - | | | - | | - | | - | | | - | - | - | | | - | | - | | - | | | - | | - | | - | - | | - | | - | | - | | | - | - | - | - | | - | - | - | |
| BOZ | | | 48 | | 19 | | | 9 | | | 4 | 5 | | | 2 | | 4 | | 3 | | | - | | - | | | 1 | | | 1 | | - | | | | - | | | - | | - | | - | | | - | - | - | | | - | | - | | - | | | - | | - | | - | - | | - | | - | | - | | | - | - | - | - | | - | - | - | |
| CAUC | | | 50 | | 14 | | | 7 | | | 3 | 3 | | | 12 | | 5 | | 1 | | | - | | - | | | - | | | - | | 1 | | | | 2 | | | - | | - | | - | | | - | - | 1 | | | - | | 1 | | - | | | - | | - | | - | - | | - | | - | | - | | | - | - | - | - | | - | - | - | |
| Ch | | | 46 | | 12 | | | - | | | 6 | 6 | | | 6 | | 8 | | - | | | - | | 5 | | | - | | | - | | - | | | | - | | | 2 | | - | | 1 | | | - | - | - | | | - | | - | | - | | | - | | - | | - | - | | - | | - | | - | | | - | - | - | - | | - | - | - | |
| Cl | | | 52 | | 9 | | | 30 | | | 6 | 3 | | | 1 | | - | | - | | | 1 | | - | | | - | | | - | | - | | | | - | | | - | | - | | - | | | - | - | - | | | - | | - | | - | | | - | | - | | - | 1 | | - | | - | | - | | | - | - | - | - | | - | - | 1 | |
| Ct | | | 66 | | 24 | | | 6 | | | 9 | 1 | | | 20 | | - | | - | | | 4 | | - | | | - | | | 1 | | - | | | | - | | | - | | - | | - | | | - | - | - | | | - | | - | | - | | | - | | - | | 1 | - | | - | | - | | - | | | - | - | - | - | | - | - | - | |
| DGL | | | 48 | | 30 | | | 2 | | | 6 | 9 | | | 1 | | - | | - | | | - | | - | | | - | | | - | | - | | | | - | | | - | | - | | - | | | - | - | - | | | - | | - | | - | | | - | | - | | - | - | | - | | - | | - | | | - | - | - | - | | - | - | - | |
| EDIL | | | 58 | | 33 | | | 1 | | | 2 | - | | | - | | - | | 4 | | | 12 | | - | | | - | | | - | | - | | | | - | | | - | | - | | - | | | - | - | 2 | | | - | | 1 | | - | | | - | | 1 | | - | - | | - | | - | | - | | | - | - | - | 1 | | 1 | - | - | |
| IV | | | 30 | | 9 | | | 1 | | | 6 | 9 | | | - | | 3 | | 1 | | | - | | 1 | | | - | | | - | | - | | | | - | | | - | | - | | - | | | - | - | - | | | - | | - | | - | | | - | | - | | - | - | | - | | - | | - | | | - | - | - | - | | - | - | - | |
| KAR | | | 30 | | 17 | | | - | | | 1 | 7 | | | - | | - | | - | | | 1 | | - | | | - | | | - | | - | | | | - | | | - | | - | | - | | | - | 1 | - | | | 1 | | - | | - | | | - | | 1 | | - | - | | - | | - | | - | | | - | 1 | - | - | | - | - | - | |
| KARM | | | 30 | | 17 | | | - | | | - | 2 | | | - | | - | | 1 | | | 4 | | - | | | - | | | - | | - | | | | - | | | 1 | | - | | - | | | 3 | - | - | | | 1 | | - | | - | | | - | | - | | - | - | | - | | - | | - | | | - | - | 1 | - | | - | - | - | |
| KRB | | | 48 | | 22 | | | 8 | | | 3 | 4 | | | - | | 1 | | 2 | | | - | | 1 | | | 3 | | | 1 | | - | | | | 1 | | | - | | - | | - | | | - | 1 | - | | | - | | - | | - | | | - | | - | | - | - | | - | | - | | 1 | | | - | - | - | - | | - | - | - | |
| KRC | | | 54 | | 29 | | | 5 | | | 4 | 8 | | | 1 | | 2 | | - | | | 1 | | 1 | | | - | | | - | | 2 | | | | - | | | - | | - | | - | | | - | - | - | | | - | | - | | - | | | 1 | | - | | - | - | | - | | - | | - | | | - | - | - | - | | - | - | - | |
| KRY | | | 46 | | 10 | | | 4 | | | 10 | 10 | | | 5 | | 2 | | 1 | | | - | | 1 | | | 1 | | | - | | - | | | | - | | | - | | - | | - | | | - | 1 | - | | | - | | - | | - | | | - | | - | | - | - | | - | | 1 | | - | | | - | - | - | - | | - | - | - | |
| KVR | | | 32 | | 6 | | | 12 | | | 4 | 2 | | | 1 | | 2 | | 2 | | | - | | - | | | - | | | 1 | | - | | | | - | | | - | | 1 | | - | | | - | - | - | | | - | | - | | - | | | - | | - | | - | - | | 1 | | - | | - | | | - | - | - | - | | - | - | - | |
| L | | | 60 | | 20 | | | - | | | 14 | 3 | | | 1 | | 17 | | 5 | | | - | | - | | | - | | | - | | - | | | | - | | | - | | - | | - | | | - | - | - | | | - | | - | | - | | | - | | - | | - | - | | - | | - | | - | | | - | - | - | - | | - | - | - | |
| LX | | | 82 | | 7 | | | 5 | | | 8 | 11 | | | 19 | | 13 | | - | | | 11 | | - | | | 1 | | | 5 | | - | | | | - | | | - | | - | | - | | | - | - | 1 | | | - | | - | | - | | | 1 | | - | | - | - | | - | | - | | - | | | - | - | - | - | | - | - | - | |
| MAN | | | 52 | | 1 | | | - | | | 4 | 11 | | | 11 | | 2 | | 6 | | | - | | - | | | 8 | | | 6 | | - | | | | 2 | | | - | | - | | - | | | 1 | - | - | | | - | | - | | - | | | - | | - | | - | - | | - | | - | | - | | | - | - | - | - | | - | - | - | |
| ME | | | 58 | | 6 | | | 15 | | | 9 | 4 | | | 4 | | 4 | | 1 | | | 1 | | 3 | | | 8 | | | - | | 2 | | | | - | | | - | | - | | - | | | - | - | - | | | - | | 1 | | - | | | - | | - | | - | - | | - | | - | | - | | | - | - | - | - | | - | - | - | |
| MNCH | | | 194 | | 55 | | | 39 | | | 16 | 36 | | | 31 | | 6 | | 1 | | | - | | 8 | | | - | | | - | | - | | | | - | | | 2 | | - | | - | | | - | - | - | | | - | | - | | - | | | - | | - | | - | - | | - | | - | | - | | | - | - | - | - | | - | - | - | |
| OL | | | 60 | | 20 | | | 3 | | | 13 | - | | | 5 | | 15 | | 1 | | | 1 | | - | | | - | | | 1 | | - | | | | - | | | 1 | | - | | - | | | - | - | - | | | - | | - | | - | | | - | | - | | - | - | | - | | - | | - | | | - | - | - | - | | - | - | - | |
| PRAM | | | 58 | | 18 | | | 6 | | | 5 | 9 | | | 3 | | 14 | | - | | | - | | - | | | 1 | | | 1 | | - | | | | - | | | - | | - | | - | | | - | - | - | | | - | | - | | - | | | - | | - | | - | - | | - | | - | | - | | | 1 | - | - | - | | - | - | - | |
| RA | | | 84 | | 14 | | | 17 | | | 10 | 9 | | | 15 | | 6 | | - | | | 1 | | 4 | | | 3 | | | 2 | | - | | | | - | | | 1 | | - | | 2 | | | - | - | - | | | - | | - | | - | | | - | | - | | - | - | | - | | - | | - | | | - | - | - | - | | - | - | - | |
| SZ | | | 52 | | 8 | | | 20 | | | 17 | 3 | | | - | | - | | 4 | | | - | | - | | | - | | | - | | - | | | | - | | | - | | - | | - | | | - | - | - | | | - | | - | | - | | | - | | - | | - | - | | - | | - | | - | | | - | - | - | - | | - | - | - | |
| VdB | | | 58 | | 17 | | | 7 | | | 8 | - | | | 10 | | 5 | | 4 | | | 2 | | - | | | 1 | | | 2 | | - | | | | - | | | - | | - | | 1 | | | - | - | - | | | - | | - | | 1 | | | - | | - | | - | - | | - | | - | | - | | | - | - | - | - | | - | - | - | |
|  | | |  | |  | | |  | | |  |  | | |  | |  | |  | | |  | |  | | |  | | |  | |  | | | |  | | |  | |  | |  | | |  |  |  | | |  | |  | |  | | |  | |  | |  |  | |  | |  | |  | | |  |  |  |  | |  |  |  | |
|  | | |  | |  | | |  | | |  |  | | |  | |  | |  | | |  | |  | | |  | | |  | |  | | | |  | | |  | |  | |  | | |  |  |  | | |  | |  | |  | | |  | |  | |  |  | |  | |  | |  | | |  |  |  |  | |  |  |  | |
|  | | |  | | **Haplotype** | | | | | | | | | | | | | | | | | | | | | | | | | | | | | | | | | | | | | | | | | | | | | | | | | | | | | | | | | | | | | | | | | | | | | | | | | | | |
| ***O. canadiensis*** | | | **2N** | | H1 | | | H2 | | | H3 | H4 | | | H5 | | H6 | | H7 | | | H8 | | H9 | | | H10 | | | H11 | | H12 | | | | H13 | | | H14 | | H15 | | H16 | | | H17 | H18 | H19 | | | H20 | | H21 | | H22 | | | H23 | | H24 | | H25 | H26 | | H27 | | H28 | | H29 | | | H30 | H31 | H32 | H33 | | H34 | H35 | H36 | |
| BIG | | | 16 | | - | | | - | | | - | - | | | - | | - | | - | | | 16 | | - | | | - | | | - | | - | | | | - | | | - | | - | | - | | | - | - | - | | | - | | - | | - | | | - | | - | | - | - | | - | | - | | - | | | - | - | - | - | | - | - | - | |
|  | | |  | |  | | |  | | |  |  | | |  | |  | |  | | |  | |  | | |  | | |  | |  | | | |  | | |  | |  | |  | | |  |  |  | | |  | |  | |  | | |  | |  | |  |  | |  | |  | |  | | |  |  |  |  | |  |  |  | |
|  | | |  | | **Haplotype** | | | | | | | | | | | | | | | | | | | | | | | | | | | | | | | | | | | | | | | | | | | | | | | | | | | | | | | | | | | | | | | | | | | | | | | | | | | |
| ***O. musimon*** | | | **2N** | | H1 | | | H2 | | | H3 | H4 | | | H5 | | H6 | | H7 | | | H8 | | H9 | | | H10 | | | H11 | | H12 | | | | H13 | | | H14 | | H15 | | H16 | | | H17 | H18 | H19 | | | H20 | | H21 | | H22 | | | H23 | | H24 | | H25 | H26 | | H27 | | H28 | | H29 | | | H30 | H31 | H32 | H33 | | H34 | H35 | H36 | |
| MUF | | | 62 | | 12 | | | 21 | | | 1 | - | | | 7 | | 3 | | 17 | | | - | | - | | | - | | | - | | - | | | | - | | | - | | - | | 1 | | | - | - | - | | | - | | - | | - | | | - | | - | | - | - | | - | | - | | - | | | - | - | - | - | | - | - | - | |
|  | | |  | |  | | |  | | |  |  | | |  | |  | |  | | |  | |  | | |  | | |  | |  | | | |  | | |  | |  | |  | | |  |  |  | | |  | |  | |  | | |  | |  | |  |  | |  | |  | |  | | |  |  |  |  | |  |  |  | |
|  | | |  | | **Haplotype** | | | | | | | | | | | | | | | | | | | | | | | | | | | | | | | | | | | | | | | | | | | | | | | | | | | | | | | | | | | | | | | | | | | | | | | | | | | |
| ***O. vignei*** | | | **2N** | | H1 | | | H2 | | | H3 | H4 | | | H5 | | H6 | | H7 | | | H8 | | H9 | | | H10 | | | H11 | | H12 | | | | H13 | | | H14 | | H15 | | H16 | | | H17 | H18 | H19 | | | H20 | | H21 | | H22 | | | H23 | | H24 | | H25 | H26 | | H27 | | H28 | | H29 | | | H30 | H31 | H32 | H33 | | H34 | H35 | H36 | |
| URI | | | 2 | | - | | | - | | | - | - | | | - | | - | | 2 | | | - | | - | | | - | | | - | | - | | | | - | | | - | | - | | - | | | - | - | - | | | - | | - | | - | | | - | | - | | - | - | | - | | - | | - | | | - | - | - | - | | - | - | - | |
|  | | |  | |  | | | | | | | | | | | | | | | | | | | | | | | | | | | | | | | | | | | | | | | | | | | | | | | | | | | | | | | | | | | | | | | | | | | | | | | | | | | |
|  | | |  | | **Haplotype** | | | | | | | | | | | | | | | | | | | | | | | | | | | | | | | | | | | | | | | | | | | | | | | | | | | | | | | | | | | | | | | | | | | | | | | | | | | |
| ***O. ammon*** | | | **2N** | | H1 | | | H2 | | | H3 | H4 | | | H5 | | H6 | | H7 | | | H8 | | H9 | | | H10 | | | H11 | | H12 | | | | H13 | | | H14 | | H15 | | H16 | | | H17 | H18 | H19 | | | H20 | | H21 | | H22 | | | H23 | | H24 | | H25 | H26 | | H27 | | H28 | | H29 | | | H30 | H31 | H32 | H33 | | H34 | H35 | H36 | |
| ARG | | | 2 | | - | | | - | | | 1 | - | | | - | | - | | 1 | | | - | | - | | | - | | | - | | - | | | | - | | | - | | - | | - | | | - | - | - | | | - | | - | | - | | | - | | - | | - | - | | - | | - | | - | | | - | - | - | - | | - | - | - | |
|  |  | | |  | | |  | |  | | | |  | | |  | | | |  | | |  | | | | |  | | |  | | |  | | | |  | |  | |  | | |  | |  | |  | | |  | |  | |  | | |  | |  | |  | | |  | |  | |  |  |  |  |  |  |  |  |  |  |  |
|  |  | | | **Haplotype** | | | | | | | | | | | | | | | | | | | | | | | | | | | | | | | | | | | | | | | | | | | | | | | | | | | | | | | | | | | | | | | | | |  |  |  |  |  |  |  |  |  |  |  |
| ***C. hircus*** | **2N** | | | Hh1 | | | Hh2 | | Hh3 | | | | Hh4 | | | Hh5 | | | | Hh6 | | | Hh7 | | | | | Hh8 | | | Hh9 | | | Hh10 | | | | Hh11 | | Hh12 | | Hh13 | | | Hh14 | | Hh15 | | Hh16 | | | Hh17 | | Hh18 | | Hh19 | | | Hh20 | | Hh21 | | Hh22 | | | Hh23 | | Hh24 | |  |  |  |  |  |  |  |  |  |  |  |
| GUA | 44 | | | - | | | - | | - | | | | - | | | - | | | | - | | | 34 | | | | | - | | | - | | | - | | | | - | | 2 | | 6 | | | 1 | | 1 | | - | | | - | | - | | - | | | - | | - | | - | | | - | | - | |  |  |  |  |  |  |  |  |  |  |  |
| GIR | 22 | | | - | | | - | | - | | | | - | | | - | | | | - | | | 17 | | | | | 2 | | | 1 | | | 1 | | | | 1 | | - | | - | | | - | | - | | - | | | - | | - | | - | | | - | | - | | - | | | - | | - | |  |  |  |  |  |  |  |  |  |  |  |
| MAL | 12 | | | - | | | - | | - | | | | - | | | - | | | | - | | | 7 | | | | | 2 | | | - | | | - | | | | - | | 3 | | - | | | - | | - | | - | | | - | | - | | - | | | - | | - | | - | | | - | | - | |  |  |  |  |  |  |  |  |  |  |  |
| ANG | 2 | | | 1 | | | - | | - | | | | - | | | - | | | | - | | | - | | | | | - | | | - | | | - | | | | - | | - | | - | | | - | | - | | - | | | - | | - | | - | | | - | | - | | - | | | - | | 1 | |  |  |  |  |  |  |  |  |  |  |  |
| DO | 2 | | | 1 | | | - | | - | | | | - | | | - | | | | - | | | - | | | | | - | | | - | | | - | | | | - | | - | | - | | | - | | - | | - | | | - | | 1 | | - | | | - | | - | | - | | | - | | - | |  |  |  |  |  |  |  |  |  |  |  |
| CRO | 16 | | | 1 | | | - | | - | | | | - | | | - | | | | - | | | 5 | | | | | - | | | - | | | - | | | | - | | - | | - | | | - | | - | | 1 | | | 1 | | 2 | | 1 | | | 1 | | 1 | | 2 | | | 1 | | - | |  |  |  |  |  |  |  |  |  |  |  |
| BCE | 16 | | | 9 | | | 3 | | 1 | | | | 1 | | | 1 | | | | 1 | | | - | | | | | - | | | - | | | - | | | | - | | - | | - | | | - | | - | | - | | | - | | - | | - | | | - | | - | | - | | | - | | - | |  |  |  |  |  |  |  |  |  |  |  |
|  | | | | | | | | | | | | | | |  | | | | | | | | | | |  | | | | | | | | | |  | | | | | | | | |  | | | | | |  | | | | | | |  | | | | | | |  | | | | | | |  | | | | |  | | | |
|  | | |  | | | | **Haplotypes** | | | | | | | | | | | |  |  |  |  |  |  |  |  |  |  |  |  |  |  |  |  |  |  |  |  |  |  |  |  |  |  |  |  |  |  |  |  |  |  |  |  |  |  |  |  |  |  |  |  |  |  |  |  |  |  |  |  |  |  |  |  |  |  |  |  |  |  |
| ***C. pyrenaica*** | | | **2N** | | | | Hp1 | | | | Hp2 | | | | Hp3 | | | |  |  |  |  |  |  |  |  |  |  |  |  |  |  |  |  |  |  |  |  |  |  |  |  |  |  |  |  |  |  |  |  |  |  |  |  |  |  |  |  |  |  |  |  |  |  |  |  |  |  |  |  |  |  |  |  |  |  |  |  |  |  |
| PYR | | | 16 | | | | 13 | | | | 2 | | | | 1 | | | |  |  |  |  |  |  |  |  |  |  |  |  |  |  |  |  |  |  |  |  |  |  |  |  |  |  |  |  |  |  |  |  |  |  |  |  |  |  |  |  |  |  |  |  |  |  |  |  |  |  |  |  |  |  |  |  |  |  |  |  |  |  |
|  | | |  | | | |  | | | |  | | | |  | | | |  | | |  | | | | |  | | |  | | | |  | | | |  |  |  |  |  |  |  |  |  |  |  |  |  |  |  |  |  |  |  |  |  |  |  |  |  |  |  |  |  |  |  |  |  |  |  |  |  |  |  |  |  |  |  |
|  | | |  | | | |  | | | |  | | | |  | | | |  | | |  | | | | |  | | |  | | | |  | | | |  |  |  |  |  |  |  |  |  |  |  |  |  |  |  |  |  |  |  |  |  |  |  |  |  |  |  |  |  |  |  |  |  |  |  |  |  |  |  |  |  |  |  |
|  | | |  | | | | **Haplotypes** | | | | | | | | | | | |  | | |  | | | | |  | | |  | | | |  | | | |  |  |  |  |  |  |  |  |  |  |  |  |  |  |  |  |  |  |  |  |  |  |  |  |  |  |  |  |  |  |  |  |  |  |  |  |  |  |  |  |  |  |  |
| ***R. rupicapra*** | | | **2N** | | | | Hr1 | | | | Hr2 | | | | Hr3 | | | |  | | |  | | | | |  | | |  | | | |  | | | |  |  |  |  |  |  |  |  |  |  |  |  |  |  |  |  |  |  |  |  |  |  |  |  |  |  |  |  |  |  |  |  |  |  |  |  |  |  |  |  |  |  |  |
| REB | | | 4 | | | | 2 | | | | 1 | | | | 1 | | | |  | | |  | | | | |  | | |  | | | |  | | | |  |  |  |  |  |  |  |  |  |  |  |  |  |  |  |  |  |  |  |  |  |  |  |  |  |  |  |  |  |  |  |  |  |  |  |  |  |  |  |  |  |  |  |
|  | | |  | | | |  | | | |  | | | |  | | | |  | | |  | | | | |  | | |  | | | |  | | | |  |  |  |  |  |  |  |  |  |  |  |  |  |  |  |  |  |  |  |  |  |  |  |  |  |  |  |  |  |  |  |  |  |  |  |  |  |  |  |  |  |  |  |
|  | | |  | | | | **Haplotypes** | | | | | | | | | | | |  | | |  | | | | |  | | |  | | | |  | | | |  |  |  |  |  |  |  |  |  |  |  |  |  |  |  |  |  |  |  |  |  |  |  |  |  |  |  |  |  |  |  |  |  |  |  |  |  |  |  |  |  |  |  |
| ***O. moschatus*** | | | **2N** | | | | Hm1 | | | | Hm2 | | | | Hm3 | | | | Hm4 | | | Hm5 | | | | | Hm6 | | | Hm7 | | | | Hm8 | | | |  |  |  |  |  |  |  |  |  |  |  |  |  |  |  |  |  |  |  |  |  |  |  |  |  |  |  |  |  |  |  |  |  |  |  |  |  |  |  |  |  |  |  |
| OMO | | | 30 | | | | 11 | | | | 5 | | | | 1 | | | | 8 | | | 1 | | | | | 1 | | | 2 | | | | 1 | | | |  |  |  |  |  |  |  |  |  |  |  |  |  |  |  |  |  |  |  |  |  |  |  |  |  |  |  |  |  |  |  |  |  |  |  |  |  |  |  |  |  |  |  |
|  | | |  | | | |  | | | |  | | | |  | | | |  | | |  | | | | |  | | |  | | | |  | | | |  |  |  |  |  |  |  |  |  |  |  |  |  |  |  |  |  |  |  |  |  |  |  |  |  |  |  |  |  |  |  |  |  |  |  |  |  |  |  |  |  |  |  |
|  | | |  | | | | **Haplotypes** | | | | | | | | | | | |  | | |  | | | | |  | | |  | | | |  | | | |  |  |  |  |  |  |  |  |  |  |  |  |  |  |  |  |  |  |  |  |  |  |  |  |  |  |  |  |  |  |  |  |  |  |  |  |  |  |  |  |  |  |  |
| ***A. lervia*** | | | **2N** | | | | Hl1 | | | | Hl2 | | | | Hl3 | | | |  | | |  | | | | |  | | |  | | | |  | | | |  |  |  |  |  |  |  |  |  |  |  |  |  |  |  |  |  |  |  |  |  |  |  |  |  |  |  |  |  |  |  |  |  |  |  |  |  |  |  |  |  |  |  |
| ALE | | | 28 | | | | 22 | | | | 5 | | | | 1 | | | |  | | |  | | | | |  | | |  | | | |  | | | |  |  |  |  |  |  |  |  |  |  |  |  |  |  |  |  |  |  |  |  |  |  |  |  |  |  |  |  |  |  |  |  |  |  |  |  |  |  |  |  |  |  |  |
|  | | |  | | | |  | | | |  | | | |  | | | |  | | |  | | | | |  | | |  | | | |  | | | |  |  |  |  |  |  |  |  |  |  |  |  |  |  |  |  |  |  |  |  |  |  |  |  |  |  |  |  |  |  |  |  |  |  |  |  |  |  |  |  |  |  |  |
|  | | |  | | | | **Haplotypes** | | | | | | | | | | | |  | | |  | | | | |  | | |  | | | |  | | | |  |  |  |  |  |  |  |  |  |  |  |  |  |  |  |  |  |  |  |  |  |  |  |  |  |  |  |  |  |  |  |  |  |  |  |  |  |  |  |  |  |  |  |
| ***B. mutus*** | | | **2N** | | | | Hm1 | | | | Hm2 | | | |  | | | |  | | |  | | | | |  | | |  | | | |  | | | |  |  |  |  |  |  |  |  |  |  |  |  |  |  |  |  |  |  |  |  |  |  |  |  |  |  |  |  |  |  |  |  |  |  |  |  |  |  |  |  |  |  |  |
| YAK | | | 26 | | | | 24 | | | | 2 | | | |  | | | |  | | |  | | | | |  | | |  | | | |  | | | |  |  |  |  |  |  |  |  |  |  |  |  |  |  |  |  |  |  |  |  |  |  |  |  |  |  |  |  |  |  |  |  |  |  |  |  |  |  |  |  |  |  |  |
|  | | |  | | | |  | | | |  | | | |  | | | |  | | |  | | | | |  | | |  | | | |  | | | |  |  |  |  |  |  |  |  |  |  |  |  |  |  |  |  |  |  |  |  |  |  |  |  |  |  |  |  |  |  |  |  |  |  |  |  |  |  |  |  |  |  |  |
|  | | |  | | | | **Haplotypes** | | | | | | | |  | | | |  | | |  | | | | |  | | |  | | | |  | | | |  |  |  |  |  |  |  |  |  |  |  |  |  |  |  |  |  |  |  |  |  |  |  |  |  |  |  |  |  |  |  |  |  |  |  |  |  |  |  |  |  |  |  |
| ***B. taurus*** | | | **2N** | | | | Ht1 | | | | Ht2 | | | |  | | | |  | | |  | | | | |  | | |  | | | |  | | | |  |  |  |  |  |  |  |  |  |  |  |  |  |  |  |  |  |  |  |  |  |  |  |  |  |  |  |  |  |  |  |  |  |  |  |  |  |  |  |  |  |  |  |
| HOL | | | 24 | | | | 20 | | | | 4 | | | |  | | | |  | | |  | | | | |  | | |  | | | |  | | | |  |  |  |  |  |  |  |  |  |  |  |  |  |  |  |  |  |  |  |  |  |  |  |  |  |  |  |  |  |  |  |  |  |  |  |  |  |  |  |  |  |  |  |
| AVI | | | 20 | | | | 15 | | | | 5 | | | |  | | | |  | | |  | | | | |  | | |  | | | |  | | | |  |  |  |  |  |  |  |  |  |  |  |  |  |  |  |  |  |  |  |  |  |  |  |  |  |  |  |  |  |  |  |  |  |  |  |  |  |  |  |  |  |  |  |
| SET | | | 10 | | | | 10 | | | | - | | | |  | | | |  | | |  | | | | |  | | |  | | | |  | | | |  |  |  |  |  |  |  |  |  |  |  |  |  |  |  |  |  |  |  |  |  |  |  |  |  |  |  |  |  |  |  |  |  |  |  |  |  |  |  |  |  |  |  |
| PAR | | | 14 | | | | 13 | | | | 1 | | | |  | | | |  | | |  | | | | |  | | |  | | | |  | | | |  |  |  |  |  |  |  |  |  |  |  |  |  |  |  |  |  |  |  |  |  |  |  |  |  |  |  |  |  |  |  |  |  |  |  |  |  |  |  |  |  |  |  |
| PIR | | | 18 | | | | 15 | | | | 3 | | | |  | | | |  | | |  | | | | |  | | |  | | | |  | | | |  |  |  |  |  |  |  |  |  |  |  |  |  |  |  |  |  |  |  |  |  |  |  |  |  |  |  |  |  |  |  |  |  |  |  |  |  |  |  |  |  |  |  |
|  | | |  | | | |  | | | |  | | | |  | | | |  | | |  | | | | |  | | |  | | | |  | | | |  |  |  |  |  |  |  |  |  |  |  |  |  |  |  |  |  |  |  |  |  |  |  |  |  |  |  |  |  |  |  |  |  |  |  |  |  |  |  |  |  |  |  |
